# Nintedanib enhances the efficacy of PD-L1 blockade by upregulating MHC-I and PD-L1 expression in tumor cells

**DOI:** 10.7150/thno.65828

**Published:** 2022-01-01

**Authors:** Jingyao Tu, Haoran Xu, Li Ma, Chunya Li, Wan Qin, Xinyi Chen, Ming Yi, Li Sun, Bo Liu, Xianglin Yuan

**Affiliations:** 1 Department of Oncology, Tongji Hospital, Tongji Medical College, Huazhong University of Science and Technology, Wuhan, China.; 2 Department of Orthopedics, Tongji Hospital, Tongji Medical College, Huazhong University of Science and Technology, Wuhan, China.

**Keywords:** Cancer immunotherapy, Nintedanib, PD-L1, MHC class I, Idiopathic pulmonary fibrosis

## Abstract

**Background:** Immune checkpoint inhibitors (ICIs), such as programmed cell death protein 1 (PD-1)/programmed death-ligand 1 (PD-L1), have been widely applied in clinical and scientific research. Despite their effective antitumor effects in clinical tumor therapy, most tumors are still resistant to ICIs and long-term benefits are lacking. In addition, tumor patients complicated with interstitial lung disease limit the application of ICI therapy. Therefore, for these cases, there is an urgent need to develop new methods to relieve lung complications and enhance the efficacy of ICI therapy. Nintedanib, a potent triple angiokinase inhibitor approved for the treatment of progressive fibrotic interstitial lung disease. However, its immunotherapy synergy properties and mechanism are still pending further exploration.

**Methods:** To explore the therapeutic potential of nintedanib and αPD-L1 combination therapy, MC38, LLC, and 4T1 tumor models were used to investigate antitumor and antimetastatic activities* in vivo*. An idiopathic pulmonary fibrosis-tumor bearing model was used to evaluate the effect of the synergy therapy on tumor model complicated with lung disease. Moreover, RNA-seq, immunohistochemistry, and flow cytometry were utilized to analyze the effect of combination treatment on the tumor microenvironment. The bioactivity following different treatments was determined by western blotting, CCK-8, and flow cytometry.

**Results:** In this study, nintedanib and αPD-L1 synergy therapy exhibited significant antitumor, antimetastatic and anti-pulmonary fibrosis effects. Both *in vitro* and *in vivo* experiments revealed that these effects included promoting vessel normalization, increasing infiltration and activation of immune cells in tumors, enhancing the response of interferon-gamma, and activating the MHC class I-mediated antigen presentation process. Moreover, our results showed an increased expression of PD-L1 and promoted phosphorylation of STAT3 after nintedanib (1 µM) treatment.

**Conclusion:** The combination of nintedanib and αPD-L1 increased ICI therapy responses, relieved lung complications and further activated the tumor immune microenvironment; thus, exhibiting a notable antitumor effect. Accordingly, the nintedanib synergy strategy is expected to be a promising candidate therapy for tumor patients complicated with interstitial lung disease in clinical practice.

## Introduction

The incidence and mortality rates of malignant tumors continue to rise and show a trend in affecting the younger population. Clinically, immunotherapy is becoming a prevalent approach that is being researched and developed for the treatment of tumors due to its evident therapeutic effects 1-4. Although immunotherapy can activate the immune system, block immune checkpoint pathways, and overcome immune escape to inhibit tumor growth, there are still many cancer patients who do not obtain long term therapeutic benefits. Among all immune checkpoint inhibitors (ICIs), programmed cell death protein 1 (PD-1)/programmed death-ligand 1 (PD-L1) has attracted the wide attention. However, only a subset of patients benefit from anti-PD-1/PD-L1 treatment, and the overall response rate is relatively low 5, 6. The effective rate of anti-PD-1/PD-L1, a novel therapy for non-small cell lung cancer (NSCLC), administered alone is approximately 20-30%, while the effective rate of conventional chemotherapy combined with immunotherapy is only 50% 7. In addition, the effectiveness of anti-PD-1/PD-L1 therapy for colon cancer 8-10 and breast cancer is still under investigation in several clinical trials 11, 12. Therefore, improving the efficacy of immunotherapy is an urgent problem that needs to be addressed in current oncology treatments.

It is well-established that many intrinsic and extrinsic factors operating within the tumor microenvironment (TME) inhibit the therapeutic effects of ICIs 13, 14. Intrinsic factors such as the absence of antigenic proteins (low mutational burden), inefficient antigen presentation (downregulation of major histocompatibility complex class I [MHC-I] or transporter associated with antigen processing [TAP]), insensitivity to T cells (oncogenic PD-L1 expression, mutations in the interferon-gamma [IFN-γ] pathway), etc., can contribute to immune escape 13, 15, 16. Extrinsic factors such as the absence of T cells, inhibitory immune checkpoints (VISTA, LAG-3, TIM-3), immunosuppressive cells (myeloid-derived suppressor cells [MDSCs], Tregs, or type II macrophages), and abnormal tumor extracellular matrix (ECM; abnormal tumor vessel and fibrotic collagen) can create an immunosuppressive environment 13, 15, 17, 18. The various factors mentioned above would eventually lead to ICI therapy failure. Therefore, dedicated research and strategies to combat these mechanisms that induce immune resistance are key to increasing the efficacy of ICI treatment.

To overcome these obstacles, strategies using synergistic anti-angiogenesis and ICI treatments have been widely utilized in preclinical studies and clinical trials with promising outcomes 19-21. However, some studies have reported that increased infiltration of immune cells is inversely related to patient overall survival and progression-free survival, which may be attributed to the fact that most of the recruited immune cells after anti-angiogenic therapy were dysfunctional 22. Therefore, elimination of immunosuppressive TME and activation of immune cells are the primary problems that need to be addressed in anti-angiogenesis treatment combined with ICI therapy.

Indeed, clinical cases have shown that tumor patients complicated with different degrees of interstitial lung disease (ILD; such as chronic obstructive pulmonary diseases, idiopathic pulmonary fibrosis [IPF], ICI therapy-related ILD and radiation-induced lung fibrosis, etc.) are often associated with poor outcomes 23. However, both ICI and anti-angiogenesis therapy may induce the occurrence or aggravation of a series of ILD 24, 25. Multiple clinical trials have shown that the incidence of ICI therapy-related ILD is approximately 3.5-14.5% after ICI therapy 26-29. In particular, the treatment of ICI therapy-related ILD is mainly based on immunosuppressive strategies, which leads to the discontinuation of ICI treatment and tumor progression 24. Thus, there is an urgent need to develop new methods to relieve lung complications and enhance the efficacy of ICI therapy.

Nintedanib, a potent triple angiokinase inhibitor (VEGFR1/2/3, FGFR1/2/3, and PDGFR α/β) 30, is known to be an excellent anti-angiogenic agent and approved for the treatment of progressive fibrotic interstitial lung disease in 2020 31-33. Moreover, recent evidence suggests that nintedanib can increase the infiltration of CD8^+^ T cells in the tumor environment and inhibit tumor proliferation by inhibiting cancer-associated fibroblasts 34, 35. Except for alleviating fibrosis in tumor ECM, we hypothesized that nintedanib normalizes distorted vessels in tumor ECM and enhances the ICI efficacy. Moreover, the mechanisms by which nintedanib acts on tumor cells to increase the immune response has not yet been reported.

On the other hand, extensive research has shown that anti-angiogenesis drugs used for tumor treatment may increase the expression of PD-L1 in tumor cells 22, 36-38. The increased expression of PD-L1 may enhance tumor sensitivity to immunotherapy (anti-PD-1/PD-L1) and improve the prognosis of patients treated with ICIs 39-44. In summary, we hypothesized that nintedanib combined with anti-PD-L1 (αPD-L1) therapy may promote immunotherapy responses and exert antitumor effects to prevent tumor progression. In this study, the MC38 and LLC tumor-bearing models were used to evaluate the antitumor effect of the synergistic therapy and an IPF-tumor bearing model was used to explore the effect of relieving lung complications. Antimetastatic abilities were verified using the 4T1 model. As for the immune landscape, we explored the difference in tumor immune microenvironment (TIME) after combination treatment with tumor tissue immunohistochemistry (IHC) and flow cytometry. To elucidate the underlying mechanisms, RNA sequencing (RNA-seq) and a series of *in vitro* experiments were performed. Through this study, we sought to clarify the mechanisms of nintedanib on TIME and explore the antitumor therapeutic potential of nintedanib combined with αPD-L1. This combination may be a potential strategy against immune escape and be applicable in clinical synergy immunotherapy.

## Methods

### Reagents and materials

Nintedanib was purchased from Selleck Chemicals (Houston, TX, USA). InVivoMab anti-mouse PD-L1 antibody (clone 10F.9G2, Cat#BE0101) was obtained from BioXcell (West Lebanon, NH, USA). Recombinant mouse interferon-gamma (rMuIFN-γ; HY-P7071) and recombinant human interferon-gamma (rHuIFN-γ; HY-P7025) were purchased from MedChem Express (Monmouth Junction, NJ, USA). Dimethyl sulfoxide was purchased from Promoter (Wuhan, China).

### Cell lines and culture

The cell lines used in this study were obtained from the Oncology Laboratory, Tongji Hospital, Huazhong University of Science and Technology. MC38 and CT26 (murine colon cancer cells), H1975, A549, H1299, H358, H292, H1703, Hcc827, PC9, and H460 (lung cancer cells) were cultured in RPMI-1640 medium (Hyclone, Logan, UT, USA), and 4T1 (murine breast cancer cells) were cultured in RPMI-1640 medium (Gibco, Grand Island, NY, USA). LLC (murine lung cancer cells), B16-F10 (murine melanoma cells), MCF-7 and 231 (breast cancer cells), and Hct-116 and Sw480 (colon cancer cells) were cultured in Dulbecco's modified Eagle's medium (Hyclone). Cells were cultured in complete medium containing 10% fetal bovine serum (Gibco) and 1% penicillin-streptomycin (Gibco), and incubated with 5% CO_2_ at 37 °C.

### Experimental animals

BALB/c mice, C57BL/6 mice (female, 6-8 weeks, 18-20 g) were obtained from Vital River Laboratories (Beijing, China). The *in vivo* experiments were performed in accordance with the guidelines of the International Guiding Principles for Animal Research and approved by the Ethics Committee of Huazhong University of Science and Technology. The mice were fed and experiments were conducted under specific pathogen-free grade conditions.

### CCK-8 assay

Cells from different cell lines were seeded into 96-well plates (5×10^3^ cells per well) and incubated at 37 °C for 24 h. Different concentrations of nintedanib were added to cells which were then cultured for 24, 48, or 72 h. At the experimental endpoint, Cell Counting Kit-8 (CCK-8; HY-K0301, MedChem Express) was used to evaluate cell viability using absorbance (wavelength of 450 nm) detected by a microplate reader (BioTek, Winooski, VT, USA).

### Western blotting

Tumor cells or tissue proteins were extracted using RIPA buffer (Beyotime, Shanghai, China) containing phenylmethylsulfonyl fluoride and phosphatase inhibitors (Servicebio, Wuhan, China), and the concentration was quantified using a BCA assay kit (Beyotime, Shanghai, China). The protein sample (30 µg) was separated using SDS-PAGE and transferred to a PVDF membrane (Millipore, Burlington, MA, USA). After blocking with 5% BSA for 1 h, the membranes were incubated with the following primary antibodies: GAPDH (1:20000, 60004-1-lg, Proteintech, Wuhan, China), TAP1 (1:800, 11114-1-AP, Proteintech), PD-L1 (1:1000, 17952-1-AP, Proteintech), PD-L1 (1:1000, E1L3N, Cell Signaling Technology, Boston, Massachusetts, USA), STAT3 (1:1000, 10253-2-AP, Proteintech), Phospho-STAT3 (1:1000, D3A7, Cell Signaling Technology), Beta-2-Microglobulin (β2M; 1:1000, 13511-1-AP, Proteintech), Fibronectin (1:1000, 15613-1-AP, Proteintech), E-cadherin (1:1000, 24E10, Cell Signaling Technology), and Vimentin (1:1000, D21H3, Cell Signaling Technology) at 4 °C overnight. The next day, membranes were incubated with secondary antibodies (Promoter, Wuhan, China) and visualized using SuperSignal West Pico Chemiluminescent Substrate (Thermo Scientific, Waltham, MA, USA). The signal from the blots was detected using the G:BOX Chemi X system (Syngene, Cambridge, UK) and analyzed using ImageJ software.

### Real-time PCR (RT-PCR)

Total tumor tissue RNA was extracted using Trizol (NO. 9766, Takara Bio, Shiga, Japan) and reverse transcribed to cDNA using the PrimeScript 1st Strand cDNA Synthesis Kit (NO.6110A, Takara Bio). Gene-specific primers used are listed: *Cxcl2*, 5'-ACTGCGCTGTCAATGCCTGAAG-3' (forward), 5'-CAGTTAGCCTTGCCTTTGTTCAGTATC-3' (reverse); *Cxcl3*, 5'-CCACCAACCACCAGGCTACA-3' (forward), 5'-TGGACTTGCCGCTCTTCAGT-3' (reverse); *Ccl6*, 5'-TTCTTTATCCTTGTGGCTGTCCTTGG‑3' (forward), 5'‑AGGCACCTCTGAACTCTCCGATC‑3' (reverse); *Ccl8*, 5'-TGCTTCTTTGCCTGCTGCTCATAG‑3' (forward), 5'‑TCTCCATGTACTCACTGACCCACTTC‑3' (reverse); *Ccl9*, 5'-TCTTCAGATTGCTGCCT GTCCTATAAC‑3' (forward), 5'‑TTGTTTGTAGGTCCGTGGTTGTGAG‑3' (reverse); *GAPDH*, 5'‑AGGTCGGTGTGAACGGATTTG-3' (forward), 5'‑TGTAGACCATGTAGTTGAGGTCA‑3' (reverse). RT-PCR was performed to detect gene expression levels using an RT-PCR system (7900HT, Applied Biosystems, Waltham, MA, USA). The mRNA expression levels of the target genes were normalized to *GAPDH* expression levels and analyzed using the ^2-ΔΔCt^ method.

### Flow cytometry

Tumor tissue, lymph nodes, and spleen were harvested from the euthanized mice (n = 5 per group). To prepare the tumor cell suspension, tumor tissue was cut into small pieces, ground, and digested with type IV collagenase (Promoter), hyaluronidase, and DNase at 37 °C. The suspensions were filtered using 70 μm nylon cell strainers and treated with RBC lysis buffer. To obtain lymph node and spleen suspensions, tissues were ground and filtered using 70 μm nylon cell strainers (spleen suspension must be processed by RBC lysis). After centrifugation (2000 rpm, 5 min) and resuspension with PBS, cells were stained with APC/Cy7 Fixable Viability Stain 780 (565388, BD, Franklin, NJ, USA), blocked with anti-mouse CD16/32 (101320, BioLegend, San Diego, CA, USA) and stained according to the manufacturer's guidelines. The fluorescent antibodies used are listed as follows: PerCP/Cy5.5-anti-mouse CD11c (560584, BD), PE/Cy7-anti-mouse CD86 (560582, BD), PE-anti-mouse CD45 (553081, BD), FITC-anti-mouse CD8a (553030, BD), BV421-anti-mouse CD4 (562891, BD), BV510-anti-mouse CD3e (563024, BD), Alexa Fluor 674-anti-mouse I-A/I-E (107608, BioLegend), APC-anti-mouse CD274 (124311, BioLegend), PE/Cy7-anti-mouse CD69 (552879, BD), FITC-anti-mouse CD11b (557396, BD), BV421-anti-mouse F4/80 (123131, BioLegend), BV650-anti-mouse CD206 (141723, BioLegend), Alexa Fluor 700-anti-mouse Ly-6G/Ly-6C (557979, BD). Flow cytometry was performed using CytoFLEX LX (Beckman, Brea, CA, USA) and analyzed using FlowJo v10 software.

### IFN-γ response analysis

Tumor cells were seeded into 6-well plates (2×10^5^ cells per well) and treated under several conditions (different concentrations of nintedanib and 10 ng/ml rMuIFN-γ) for 24 h. A total of 5×10^5^ cells were obtained, washed with PBS, and stained with fluorescent antibodies: APC-anti-mouse H-2Kd (116619, BioLegend), FITC-anti-mouse H-2Kb (553569, BD), and PE-anti-mouse CD274 (558091, BD). Flow cytometry was performed and analyzed as described above.

### RNA‑seq assay

The RNA-seq assay was performed by Novogene (Beijing, China). Briefly, the total RNA of MC38 tumors (minimum of four samples per group) was extracted using Trizol (Takara Bio) and purified for library preparation and sequencing on an Illumina Hiseq platform. For data analysis, differential expression analysis of the four groups was performed using the DESeq2 R package (1.20.0), and genes were considered significantly differentially expressed when the* p*-value was less than 0.05. Gene Ontology (GO) and Kyoto Encyclopedia of Genes and Genomes (KEGG) pathway analyses for differentially expressed genes (DEGs) were performed using the clusterProfiler R package. Immune signatures were designed based on the public lists 45. In addition, we used the local Gene Set Enrichment Analysis (GSEAtool (http://www.broadinstitute.org/gsea/index.jsp) to independently analyze the GO and KEGG data sets.

### Staining and IHC

Isolated lung tissues and tumors were fixed with 4% paraformaldehyde (PFA) at 25 °C for 48 h, dehydrated, and embedded in paraffin wax for further experiments. Hematoxylin-eosin (HE) staining (G1005, Servicebio), Masson's trichrome staining (G1340, Solarbio, Beijing, China) and various IHC assays were performed according to the manufacturer's instructions. The primary antibodies used in the experiments were as follows: anti-CD3 (1:150, ab16669, Abcam, UK), anti-CD8α (1:2000, ab217344, Abcam), and anti-Ki67 (1:200, ab16667, Abcam). Bright-field images (five random fields) were captured using a microscope (EVOS fl auto, Thermo Fisher Scientific, USA). For CD3^+^ and CD8^+^ T cell analysis, two blinded pathologists counted tumor center and tumor periphery T cells. In addition, positive Ki67 cells were counted and analyzed using ImageJ software.

### Immunofluorescence (IF)

Freshly dissected tumor tissues were fixed with 4% PFA, dehydrated, embedded in paraffin wax, and transferred to the slice. A TUNEL assay staining kit (11684817910, Roche) was used to detect tumor cell apoptosis. Next, according to the manufacturer's protocol, the slices were permeabilized (0.1% Triton X-100), blocked with 10% normal donkey serum (ANT051, antGene, Wuhan, China), and incubated with primary antibodies, CD31 (1:500, ab182981, Abcam), and α-SMA (1:100, AF1032, Affinity) overnight. After incubation with fluorescent secondary antibody and DAPI (C0065, Solarbio), the immunofluorescence images were captured using a microscope (EVOS fl auto, Thermo Fisher Scientific, Waltham, MA, USA) and analyzed using ImageJ software.

### Tumor growth inhibition

For *in vivo* experiments, two different subcutaneous tumor models (MC38 and LLC) were established by subcutaneous inoculation of tumor cells (1×10^6^) into the right flank of C57BL/6 mice. When the volume of the tumor was approximately 100 mm^3^, the mice (n = 7) were randomly treated with isotype control antibodies (*i.p.*) and saline (*p.o.*) as the control group, αPD-L1 (*i.p.*, 10 mg/kg, three times a week, administered three times) as the αPD-L1 group, nintedanib (*p.o.*, 30 mg/kg, 5 days ON, 1 day OFF, administered 10 times) as the nintedanib group, or a combination of αPD-L1 with nintedanib as the combination group. Subsequently, tumor size (length × width^2^ / 2) and mouse weight were recorded every three days. The endpoint of the animal experiment was when a tumor volume from one mouse was > 2000 mm^3^ or when natural death of the mice occurred. Finally, the tumor volume, tumor weight, and mouse body weight were measured, and tumor tissues were collected for further experiments.

Survival and tumor growth curves of the tumor-bearing mice were investigated in an independent experiment. Two tumor models were grouped and treated as described above (n = 7). Mouse tumor volumes were recorded until all mice reached the endpoint and were analyzed.

### Tumor metastasis inhibition

To validate the antimetastatic effect, a metastatic 4T1 tumor model was established by transplanting 4T1 cells (1×10^6^) into the mammary fat pad of Balb/c mice. When the tumor volume reached 50-80 mm^3^, the tumor-bearing mice were grouped and treated as mentioned above in the “Tumor growth inhibition” subsection. At the end of the experiment (tumor volume > 2000 mm^3^), the mice were euthanized. During necropsy, the tumor and lung tissues were weighed and dissected for further investigation. In addition, the lung tissues were fixed with Bouin's fluid to display and count the metastatic nodules.

### IPF-tumor bearing model

IPF model mice were anesthetized by isoflurane and established by single intratracheal administration of Bleomycin sulfate (1.5 mg/kg, HY-17565, MedChem Express) via microsprayer aerosolizer. After 7 days, MC38-luc tumor cells (1×10^6^) were injected subcutaneously into the right flank of IPF model. When the volume of the tumor was approximately 100 mm^3^, the mice were divided into control, αPD-L1 and combination group randomly (n = 12) and received corresponding treatment (Figure 8A). Survival of mice were monitored daily. Tumor growth is reflected by volume measurement every three days and *in vivo* fluorescence imaging on day 10 after treatment. At the endpoint, mice pulmonary fibrosis were evaluated by micro CT scan, HE and Masson's trichrome staining 46.

### *In vivo* fluorescence imaging

Mice were injected by D-Luciferin (115144-35-9, GoldBio, USA) and anesthetized with pentobarbital sodium (50 mg/kg). Bioluminescence was detected by IVIS 200 Xenogen system (IVIS Spectrum; Perkin Elmer, Waltham, USA).

### Micro CT scan

Mice were anesthetized with pentobarbital sodium and set in the prone position. Thorax computed tomography (CT) was scanned and reconstructed by micro-CT scanner (SkyScan 1176, Bruker, Billerica, MA, USA). The IPF model lung density was quantified as Hounsfield units (HU) used in clinical CT scanners.

### Safety evaluation

At the end of the animal experiment, the main organs (heart, liver, lung, colon, spleen, and kidney) and serum of the euthanized mice were collected for further investigation. Major organs were fixed with 4% PFA, embedded in paraffin, and sectioned for HE staining analysis to evaluate histopathological damage. Mouse serum was collected for blood biochemistry evaluation (alanine aminotransferase [ALT], aspartate aminotransferase [AST], blood urea nitrogen [BUN], and creatinine [CRE]) and analyzed using a Chemray 240 Automatic Biochemical Analyzer (Rayto Science, Shenzhen, China).

### Statistical analyses

All statistical analysis and statistical graph generation were performed using GraphPad Prism 8, and the data are presented as mean ± standard deviation. Student's t-test was performed to analyze data between two experimental groups, while multiple group comparisons were performed using one-way analysis of variance (ANOVA). Statistical significance was shown as *p < 0.05, **p < 0.01, ***p < 0.001, ****p< 0.0001.

## Results

### Antitumor ability of nintedanib combined with αPD-L1 *in vivo*

**Figure 1:**
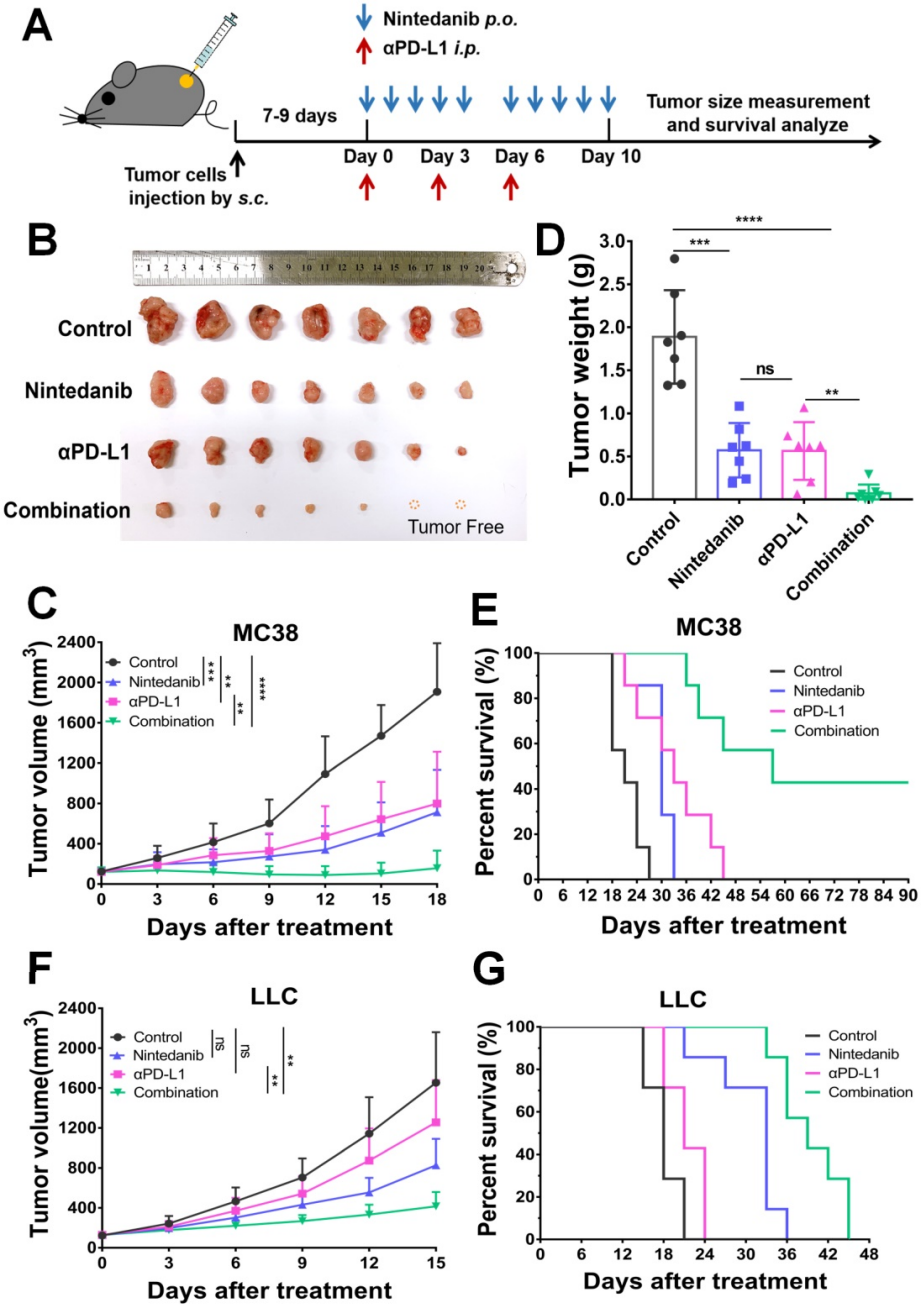
** The antitumor effect of nintedanib combined with α-programmed death-ligand 1 (αPD-L1) *in vivo*. (A)** Treatment schedule of tumor-bearing mice. **(B)** Images of isolated tumors from MC38 tumor-bearing mice. **(C)** Growth curves and **(D)** weight of tumors from MC38 tumor-bearing mice. **(E)** Survival curves of MC38 tumor-bearing mice. **(F)** Tumor growth curves and **(G)** survival curves of LLC tumor-bearing mice.

The antitumor effect of nintedanib combined with αPD-L1 was evaluated in both the MC38 and LLC tumor models. After subcutaneous injection of tumor cells for 7-9 days (tumor volume around 100 mm^3^), mice were grouped and treated as shown in Figure 1A (“Tumor growth inhibition” subsection of the Methods). Although the nintedanib and αPD-L1 groups partially suppressed tumor growth in the MC38 tumor model, the combination therapy exhibited a notable antitumor effect, with some tumors (2/7) showing complete regression (Figure 1B). Individual tumor growth curves are shown in Figure S1A. The tumor volume curves and tumor weight also reflected the tumor suppression effect of the combination therapy (Figure 1C-D). Furthermore, the combination group showed prolonged overall survival compared to the other groups (Figure 1E). Individual tumor growth curves are shown in Figure S1B. In the LLC tumor model, αPD-L1 monotherapy did not slow tumor progression and reduce tumor burden, which may be due to the weak response of LLC tumors to immune checkpoint therapy (Figure 1F). However, αPD-L1 combined with nintedanib effectively inhibited tumor growth and prolonged overall survival of the mice compared to monotherapy (Figure 1F-G, and Figure S1C), demonstrating the enhanced immunotherapy effect of nintedanib.

### Nintedanib promotes tumor cell apoptosis and tumor blood vessel normalization

**Figure 2:**
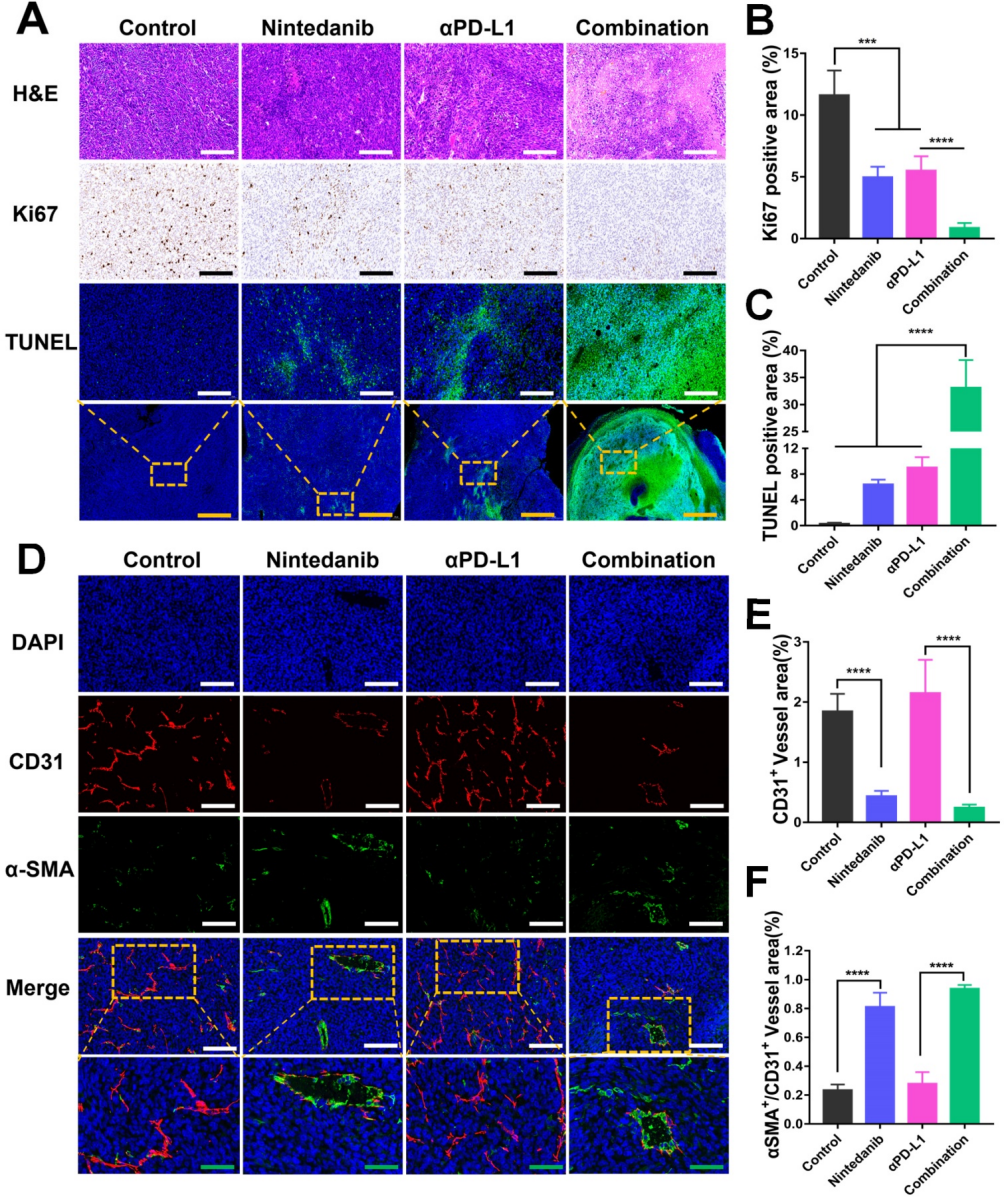
** Image analysis of tumor tissue stained with different stains. (A)** Images of tumor tissue stained with hematoxylin-eosin (HE), Ki67, and TUNEL. Quantitative analysis of the **(B)** Ki67 positive area and **(C)** TUNEL positive area. **(D)** Immunofluorescence (IF) images of CD31 and α-SMA in LLC tumor tissue. The quantitative analysis of the **(E)** CD31^+^ vessel area and **(F)** α-SMA^+^/ CD31^+^ vessel area. The green scale bar is 100 µm, the white and black scale bar is 200 µm, and the yellow scale bar is 1000 µm.

Tumors isolated from the MC38 and LLC models that received different treatments were collected for further investigation. As shown in Figure 2A-C, the promotion of tumor apoptosis and inhibition of tumor proliferation were confirmed with HE, Ki67, and TUNEL staining. Relative to the control, nintedanib, and αPD-L1 groups, the combination group showed the highest necrotic and apoptotic activity and the largest population of TUNEL-positive cells, while Ki67 had the lowest level of expression in this group. A biomarker of angiogenesis, CD31, used to monitor vessel density was detected in tumor tissues by IF analysis. The mean vessel density in tumors was notably reduced after nintedanib administration (Figure 2D-E, and Figure S2). In contrast, α-SMA^+^/CD31^+^ (a sign of tumor vessel normalization), which reflects perfused functional tumor vessels, was markedly increased (Figure 2D-F, and Figure S2). From this it can be concluded that dying tumor cells and functional blood vessel normalization may contribute to the infiltration and activation of immune cells.

### Nintedanib increases immune cell infiltration and reshapes the TIME

**Figure 3:**
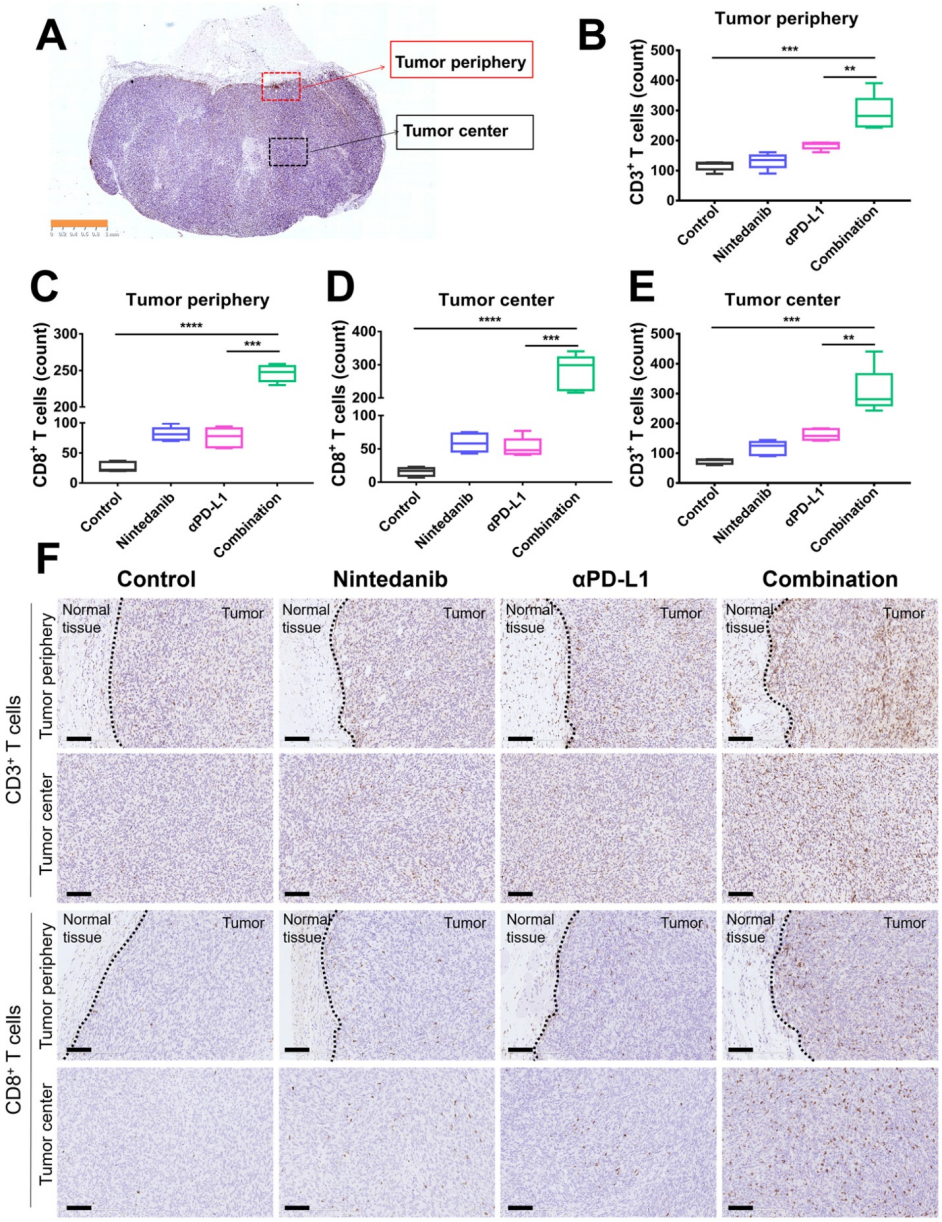
** Immunohistochemistry (IHC) analysis of CD3^+^ T cells and CD8^+^ T cells in MC38 tumor tissues. (A)** Schematic diagram of the tumor center and periphery. Quantitative analysis of **(B)** CD3^+^ T cells and **(C)** CD8^+^ T cells in the tumor periphery. Quantitative analysis of **(D)** CD8^+^ T cells and **(E)** CD3^+^ T cells in the tumor center. **(F)** IHC images of CD3^+^ T cells and CD8^+^ T cells in the tumor periphery and center. The scale bar is 100 µm.

To further determine the immune landscape differences of synergistic nintedanib and αPD-L1 therapy, IHC of CD3^+^ and CD8^+^ T cells in MC38 tumors was performed. CD3^+^ and CD8^+^ T cells in the tumor center and tumor periphery were counted and analyzed (Figure 3A). IHC images (Figure 3F) and quantification (Figure 3B-E) prove that combination treatment remarkably facilitated the infiltration of CD3^+^ and CD8^+^ T cells into both the tumor center and the tumor periphery.

**Figure 4:**
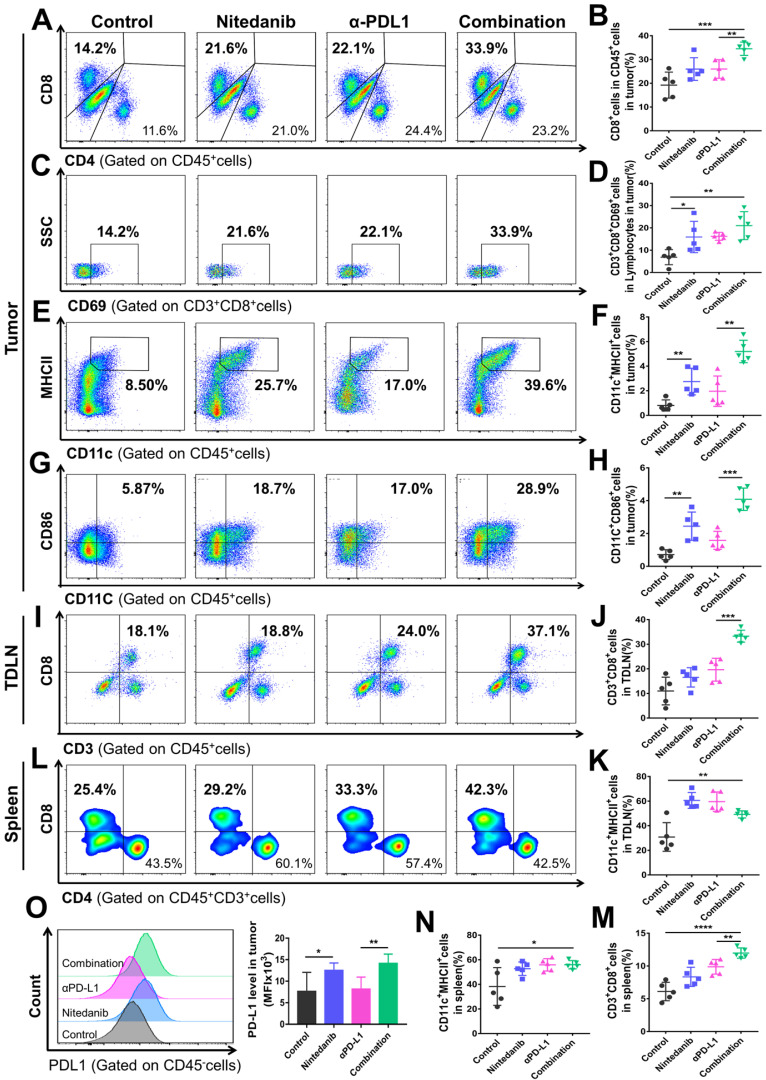
** Flow cytometry analysis of the immune microenvironment in MC38 tumor tissue, tumor-draining lymph node (TDLN), and spleen.** Representative images of flow cytometry of **(A)** CD8^+^ T cells (gated on CD45^+^ cells), **(C)** CD69^+^ T cells (gated on CD3^+^CD8^+^ cells), **(E)** CD11c^+^MHCII^+^ cells (gated on CD45^+^ cells), and **(G)** CD11c^+^CD86^+^ cells (gated on CD45^+^ cells). Quantitative analysis of the proportion of **(B)** CD8^+^ cells in CD45^+^ cells, **(D)** CD3^+^CD8^+^CD69^+^ cells in lymphocytes, **(F)** CD11c^+^MHCII^+^ cells, and **(H)** CD11c^+^CD86^+^ cells in tumors. Representative images of flow cytometry of **(I)** CD3^+^CD8^+^ T cells (gated on CD45^+^ cells) and quantitative analysis of the proportion of **(J)** CD3^+^CD8^+^ T cells and **(K)** CD11c^+^MHCII^+^ cells in the TDLN. Representative images of flow cytometry of **(L)** CD8^+^ T cells (gated on CD45^+^CD3^+^ cells) and quantitative analysis of the proportion of **(M)** CD3^+^CD8^+^ T cells and **(N)** CD11c^+^MHCII^+^ cells in the spleen. Representative images of flow cytometry of **(O)** the PD-L1 level (gated on CD45^-^ cells) and quantitative analysis of PD-L1 mean fluorescence intensity (MFI) in tumors.

The tumor tissue, spleen, and tumor-draining lymph node (TDLN) of the MC38 and LLC models were collected to explore the immune landscape using flow cytometry. In the MC38 model, combination treatment significantly promoted CD8^+^ T cell infiltration and activation in the tumor tissue (Figure 4A-D). In addition to T cells, the combination treatment also increased the density of dendritic cells (DCs) and promoted their activation simultaneously (Figure 4E-H). Nintedanib alone partially enhanced the infiltration and activation of immune cells compared to the control group. As for changes in the TDLN and spleen immune microenvironment, after combination treatment, we found that the density of CD8^+^ T cells also increased notably, while the DC number was similar to that of the monotherapy group but more than that of the control group (Figure 4I-N). Due to their recruitment in tumor tissue, the number of DCs after combination treatment did not further increase in the TDLN and spleen compared to nintedanib or αPD-L1 monotherapy. Moreover, PD-L1 levels in tumor cells were increased after nintedanib alone and the combination treatment (Figure 4O).

A similar tendency was observed in the LLC model flow cytometry results. After combination treatment, the TIME not only showed an enhancement in total lymphocyte cells, CD45^+^ cells, CD8^+^ T cells, and DC infiltration and activation (Figure S3A-F and S3K-M), but also a notable reduction in immunosuppressive MDSCs (Figure S3G-H). Although there was no significant difference in the total number of tumor-associated macrophages (TAMs), the combination group regulated the polarization of macrophages and increased the ratio of M1-like/M2-like macrophages (M1/M2; Figure S3I-J, N). In addition, increased CD8^+^ T cell density and activation was also detected in the LLC model TDLN and spleen (Figure S4). Our results collectively demonstrated that the combination of nintedanib and αPD-L1 could effectively promote T cell and DC infiltration and activation, while eliminating the immunosuppressive environment (MDSCs and TAMs ratio), thus enhancing the antitumor immune response.

### Nintedanib combined with αPD-L1 further activates immune-related pathways

**Figure 5:**
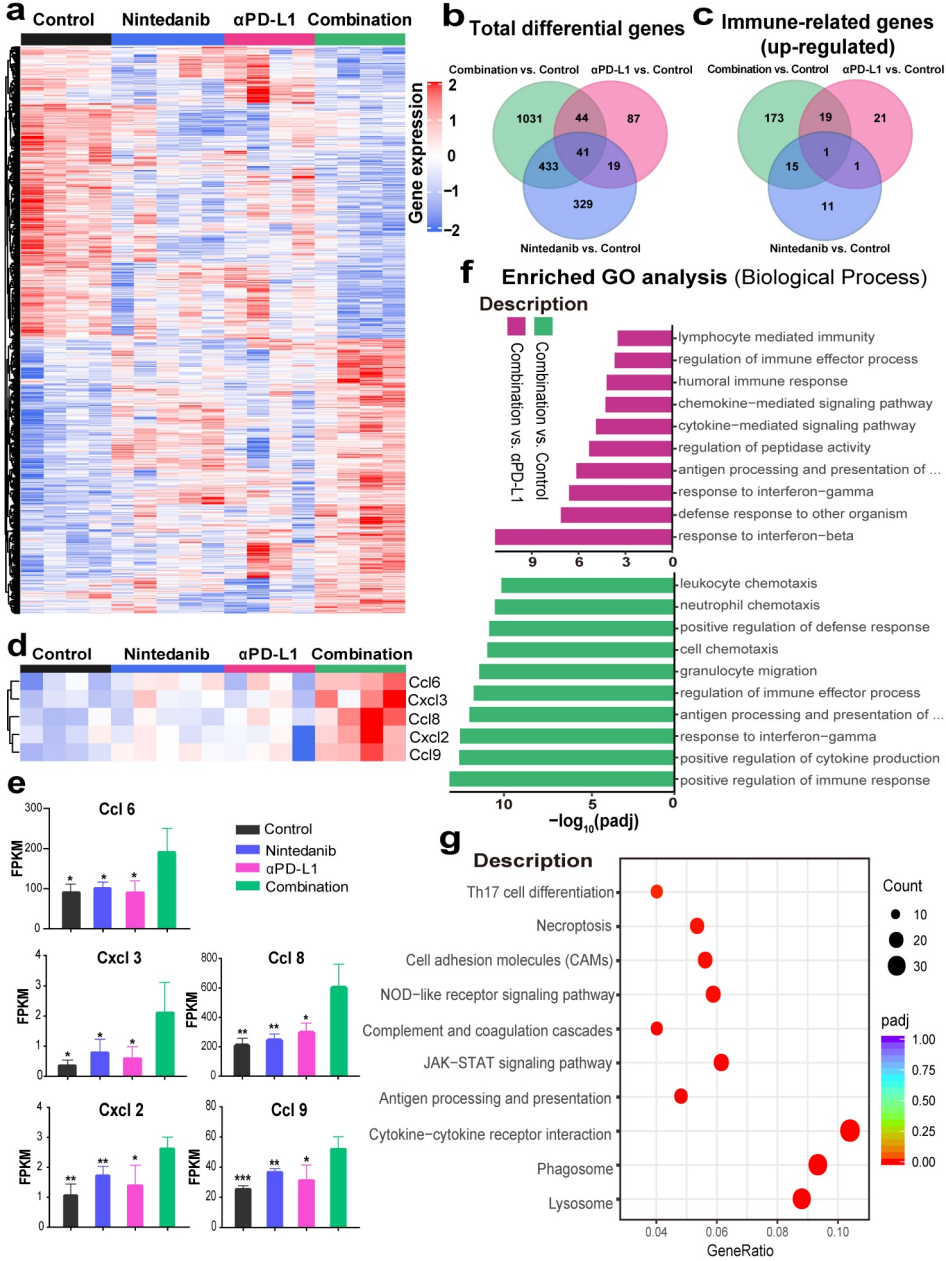
** RNA sequencing (RNA-seq) to explore changes in the immune microenvironment of MC38 tumors after different treatments. (A)** Heat map of genes that were significantly differentially expressed (*p* < 0.05). **(B)** Venn diagram of total differentially expressed genes (DEGs). **(C)** Venn diagram of the top 100 immune-related Gene Ontology (GO) terms union for the DEGs. **(D)** The expression levels of *CcL6*, *Ccl8*, *Ccl9*, *Cxcl2* and *Cxcl3,* and their **(E)** fragments per kilobase million (FPKM) quantification. **(F)** The top 10 significantly enriched immune-related GO terms. **(G)** The Kyoto Encyclopedia of Genes and Genomes (KEGG) dot plot (combination vs. control group).

To investigate the mechanisms of the enhancement of the antitumor effects and the increase of immune cell infiltration after synergistic treatment, RNA-seq in MC38 model tumor tissues was performed. DEG analysis revealed that 1549, 291, and 349 genes were differentially expressed in the control, nintedanib, and αPD-L1 groups, respectively, relative to the combination group (Figure 5A). The total DEGs are also presented intuitively in a Venn diagram (Figure 5B). Among the top 100 immune-related GO terms union for the DEGs, the combination group showed obvious upregulation of immune-related genes compared to other therapies (Figure 5C). A detailed pathway and gene list are shown in Table S1. Among these, some immune-chemokine-related genes, such as *Ccl6*, *Ccl8*, *Ccl9*, *Cxcl2,* and *Cxcl3*, were significantly upregulated after combination treatment (Figure 5D-E), which was verified with RT-PCR (Figure S5). The functional classification and enrichment of the RNA-seq results was performed using GO and KEGG analysis. The top 10 significantly (*p* < 0.05) enriched immune-related GO terms for the DEGs in the combination group compared to the control or αPD-L1 groups are shown in Figure 5F and Table S2. Combination therapy activated immune-related pathways (such as response to IFN-γ, antigen processing and presentation of peptide antigen via MHC-I, etc.) to a larger degree than αPD-L1 monotherapy. In addition, KEGG analysis of the combination and control groups also reflected the activation of the immune system (such as JAK-STAT signaling pathway, antigen processing and presentation, and cytokine-cytokine receptor interaction, etc.) in the combination group (Figure 5G). The RNA-seq results may provide further evidence for nintedanib improving the antitumor effects of ICI therapy and increasing immune cell infiltration in the transcriptomic level.

### Nintedanib enhances IFN-γ response and the MHC-I-mediated antigen presentation process

**Figure 6:**
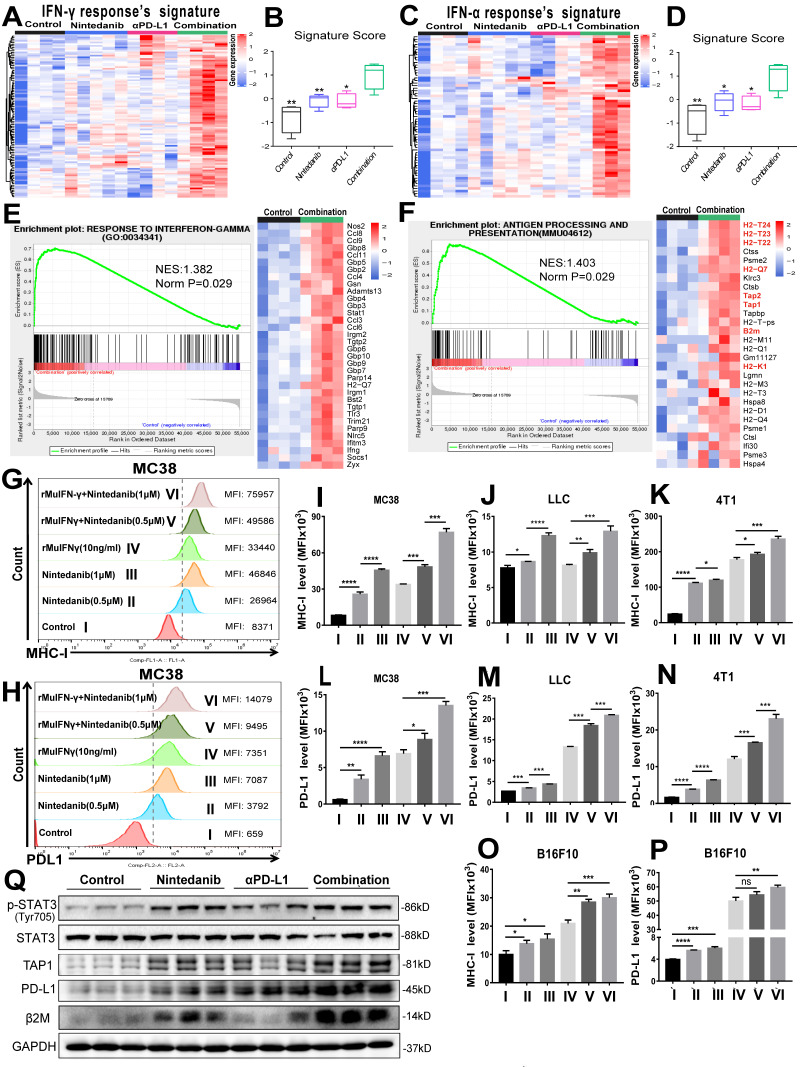
** Analysis of mechanisms related to immune activation. (A)** Heat map of the expression of signature genes of interferon-gamma (IFN-γ) response **(B)** quantified by signature score. **(C)** Heat map of the expression of signature genes of IFN-α response **(D)** quantified by signature score. Gene Set Enrichment Analysis (GSEA) plot and gene sets for **(E)** RESPONSE TO INTERFERON-GAMMA and **(F)** ANTIGEN PROCESSING AND PRESENTATION. Flow cytometry analysis of **(G)** major histocompatibility complex class I (MHC-I) and **(H)** PD-L1 expression after different treatments in MC38 cells. The MHC-I expression levels were evaluated by MFI in **(I)** MC38, **(J)** LLC, **(K)** 4T1, and** (O)** B16F10 cells. The PD-L1 expression levels were evaluated by MFI in **(L)** MC38, **(M)** LLC, **(N)** 4T1, and **(P)** B16F10 cells. **(Q)** Representative western blot images of p-STAT3/STAT3, transporter associated with antigen processing 1 (TAP1), PD-L1 and beta-2-microglobulin (β2M).

To determine the effects of different treatments on tumor cells, we screened and analyzed immune-related signature gene sets that directly affect immune response. As shown in Figure 6A-D, enriched genes and signature scores of IFN-γ and IFN-α response were increased after combination treatment. A detailed list of the enriched genes is presented in Tables S3 and S4. Additionally, GSEA also showed that genes in the combination group were significantly enriched in “RESPONSE TO INTERFERON-GAMMA” (Figure 6E) and “ANTIGEN PROCESSING AND PRESENTATION” (Figure 6F). Among the genes enriched in the latter category were *H2-K1*, *H2-T24*, *H2-T23*, *H2-T22*, *H2-Q7*, *TAP 1/2* and *β2M*, which are closely related to MHC-I, a key player in antigen processing and presentation. Based on the screening and experimental results mentioned above, we hypothesized that nintedanib could improve the efficacy of immunotherapy by enhancing the IFN-γ response and upregulating MHC-I expression in tumor cells.

To test our hypothesis, flow cytometry analysis of different tumor cell lines was performed (Figure S6A). Although rMuIFN-γ treatment partially increased MHC-I, rMuIFN-γ + nintedanib (0.5 μM or 1 μM) markedly upregulated MHC-I expression, which was seen with an increase in mean fluorescence intensity (MFI; Figure 6G, I-K, O and Figure S6B). In response to rMuIFN-γ - a stimulating factor that activates PD-L1 expression - PD-L1 expression was upregulated to a certain degree. However, PD-L1 expression was further upregulated when IFN-γ and nintedanib were combined (Figure 6H, I-N, P and Figure S6B). These results indicated that nintedanib enhanced the tumor cell response to IFN-γ, thus upregulating MHC-I and PD-L1 expression. However, in the CT26 cells (Figure S6C-D), this synergistic treatment only increased PD-L1 expression and not MHC-I expression.

At the protein level, the upregulation of p-STAT3, TAP1, β2M (MHC-I-related protein) and PD-L1 were also observed in MC38 tumor tissues (Figure 6Q). In *in vitro* experiments, a low concentration of nintedanib (1 μM) can promote STAT3 phosphorylation in different tumor cell lines (Figure S7A). Similarly, PD-L1 and β2M were upregulated in tumor cells under nintedanib (1 μM) treatment, while TAP1 had no obvious effect (Figure S7B). In addition, nintedanib combined with IFN-γ could further upregulate TAP1, PD-L1 and β2M expression compared with a single intervention (Figure S7C). IFN-γ exerted its action in TME mainly by regulating the STAT1 pathway 47, 48. Therefore, nintedanib combined with IFN-γ further activated downstream protein expression which may be attributed to additional STAT3 pathway activation.

Previously, we explored PD-L1 protein levels in various human cancer cell lines (Figure S7D) and low levels of PD-L1 were found in colon cancer cell lines and breast cancer cell lines, which may cause αPD-L1 therapy resistance. Thus, nintedanib may be an ideal complementary therapy for these tumor immunotherapies. Additionally, there were no obvious cytotoxicity of nintedanib on the viability of different tumor cells and human umbilical vein endothelial cells (HUVECs) at the experimental concentration (≤ 1 μM) within 24 h, except for the H1703 cell line (Figure S8 and S9). Apart from a series of positive effects on the TME, nintedanib increased the PD-L1 level and enhanced the αPD-L1 response, thereby exerting an excellent antitumor effect.

### Nintedanib inhibits tumor metastasis

**Figure 7:**
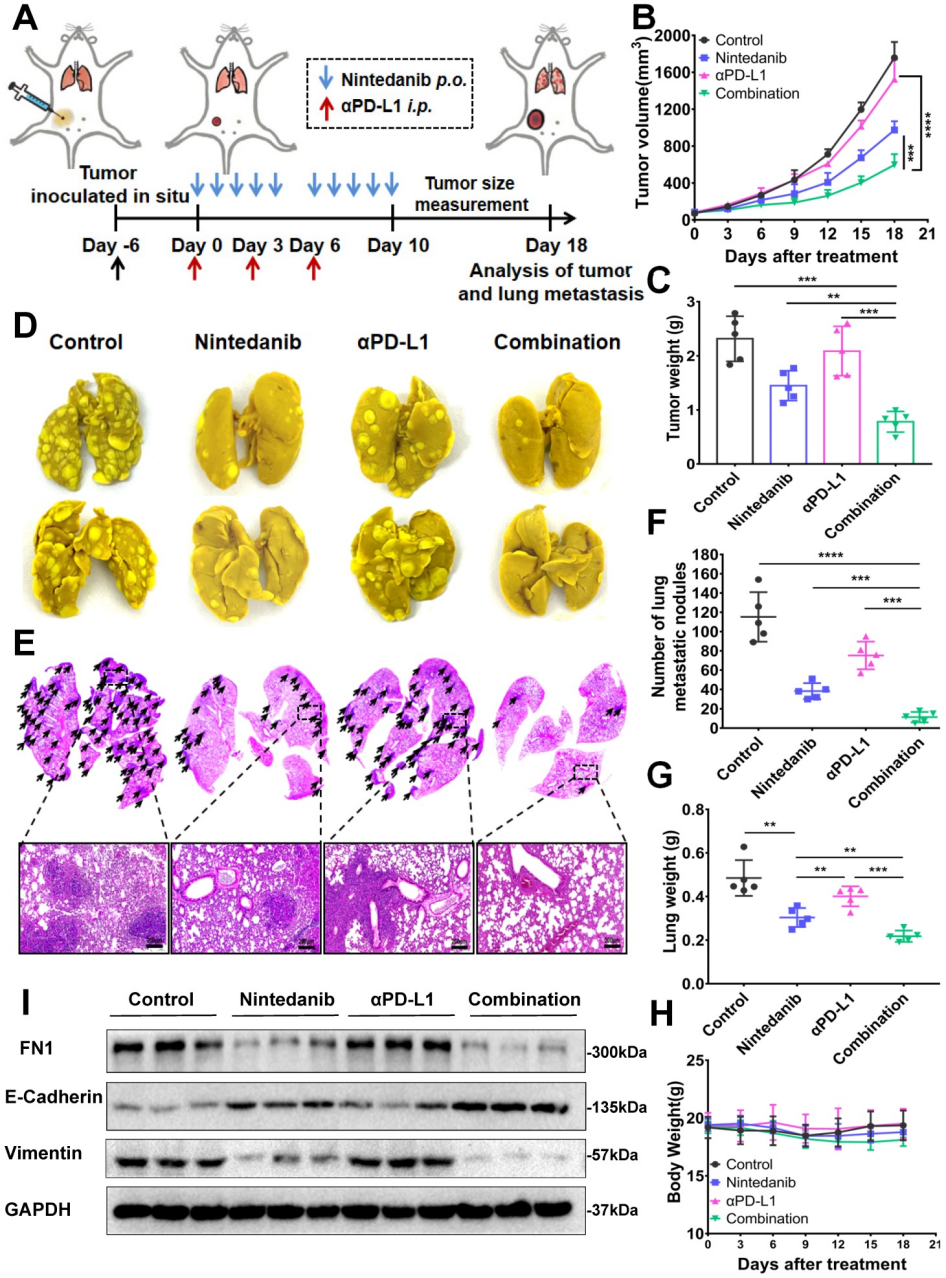
** The antimetastatic effect of nintedanib combined with αPD-L1. (A)** Treatment schedule of 4T1 tumor-bearing mice. **(B)** Growth curves and **(C)** weight of tumors from 4T1 tumor-bearing mice. **(D)** Images and **(E)** HE staining of lung tissues and lung metastatic nodules (black arrows are lung metastatic nodules). **(F)** Count of lung metastatic nodules. **(G)** Lung and **(H)** body weight of 4T1 models. **(I)** Representative western blot of epithelial-mesenchymal transition (EMT) related indicators. The scale bar is 200 µm.

Nintedanib, which enhanced the efficacy of immunotherapy, also inhibited tumor metastasis in a metastatic 4T1 tumor model. Establishment of the metastatic 4T1 tumor model (Figure S10A), and the medication method is shown in Figure 7A. The combination group notably inhibited the growth of 4T1 tumors compared to the effect on tumor growth in the other groups (Figure 7B-C, and Figure S10B-C). As presented in Figure 7D-G and Figure S10D-E, there were obvious metastatic nodules in the lung tissue of the control group and the αPD-L1 group, whereas the nintedanib-treated group had fewer nodules, suggesting that it potentially has antitumor and antimetastatic effects. Moreover, the combination of nintedanib and αPD-L1 demonstrated further anti-invasive capabilities without increasing the physical burden (Figure 7H). The mechanism by which nintedanib inhibits tumor metastasis may be related to the reversal of the epithelial-mesenchymal transition (EMT) in the tumor microenvironment. Mesenchymal protein (Vimentin, FN) levels increased while epithelial protein (E-cadherin) levels were decreased in 4T1 tumor tissue (Figure 7I). In addition, *in vitro* experiments, similar EMT reversal trend could be observed in different tumor cells after nintedanib treatment (Figure S10F). The metastatic tumor signature EMT tendency was reversed after nintedanib treatment, which may contribute to increased immune cell infiltration and enhanced antitumor effects and metastasis inhibition 49, 50. These results demonstrate that there are more options for the treatment of metastatic tumors.

### Nintedanib alleviates pulmonary fibrosis and increases the efficacy of immunotherapy in IPF-tumor model

**Figure 8:**
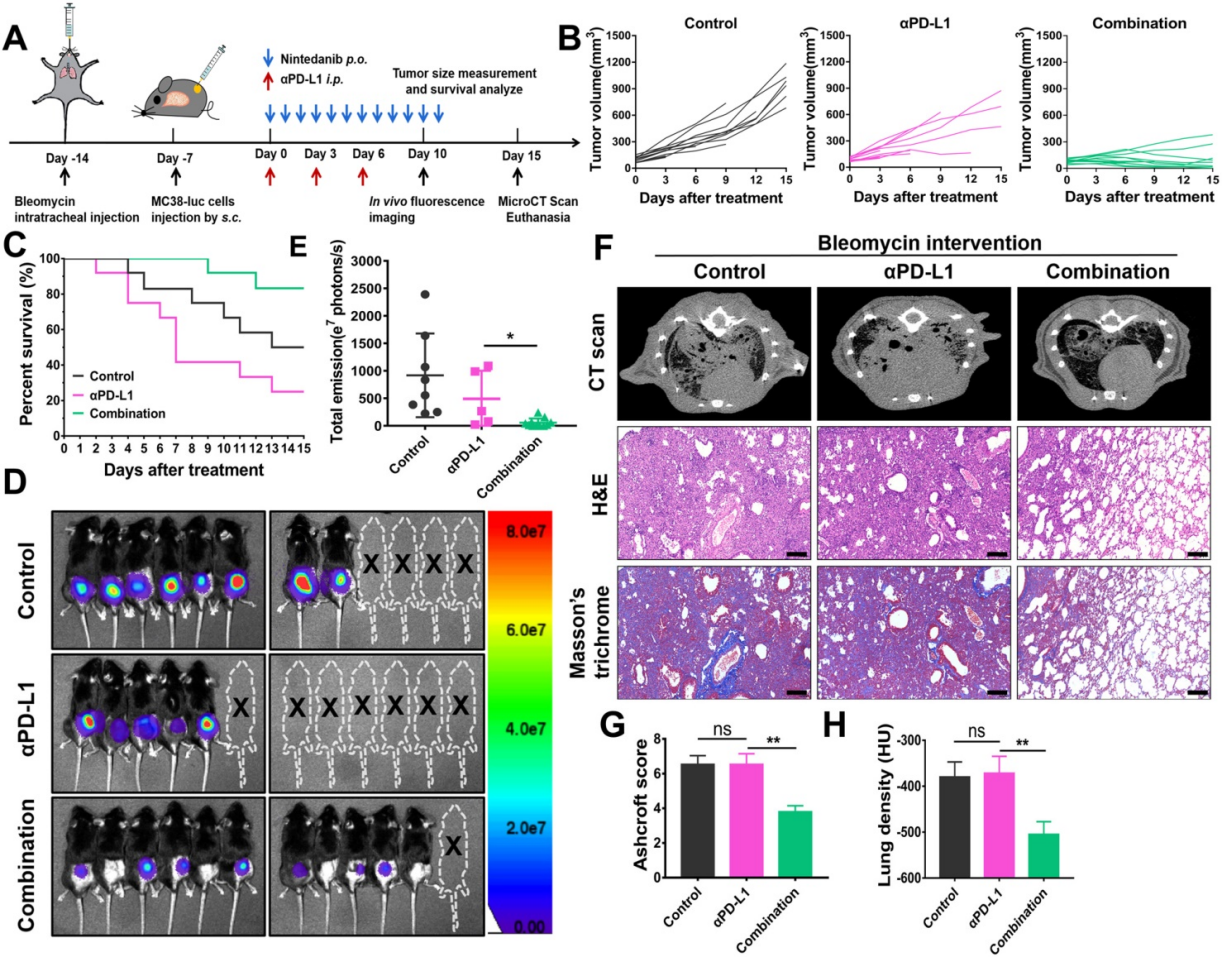
** The effects of nintedanib combined with αPD-L1 on the IPF-tumor model. (A)** Model establishment and treatment schedule of IPF-tumor model. **(B)** Individual tumor volume curves for the IPF-tumor model. **(C)** Survival curves of the IPF -tumor model. **(D)** *In vivo* fluorescence imaging and **(E)** its quantitative analysis on day 10. **(F)** The mirco CT scan, HE and Masson's trichrome staining of mice lungs. **(G)** CT lung density quantification and **(H)** lung Ashcroft score. The scale bar is 200 µm.

To mimic tumor patients complicated with lung disease, IPF-tumor bearing model was established and administered as shown in Figure 8A. Under IPF conditions, combination therapy significantly inhibits tumor growth compared to other treatments (Figure 8B, 8D-E). Although combination therapy effectively improves the survival rate of the model, mice receiving αPD-L1 alone had higher mortality than those in the control group (Figure 8C-D). As shown in lung tissue staining and CT scan results, lungs of mice in the control and αPD-L1 groups had evident pulmonary consolidation, whereas nintedanib application notably reduced lung fibrosis (Figure 8F-H). These findings suggest that the combination of nintedanib and αPD-L1 may be applied as a potential treatment for tumor patients complicated with ILD.

### Nintedanib combined with αPD-L1 is found safe

**Figure 9:**
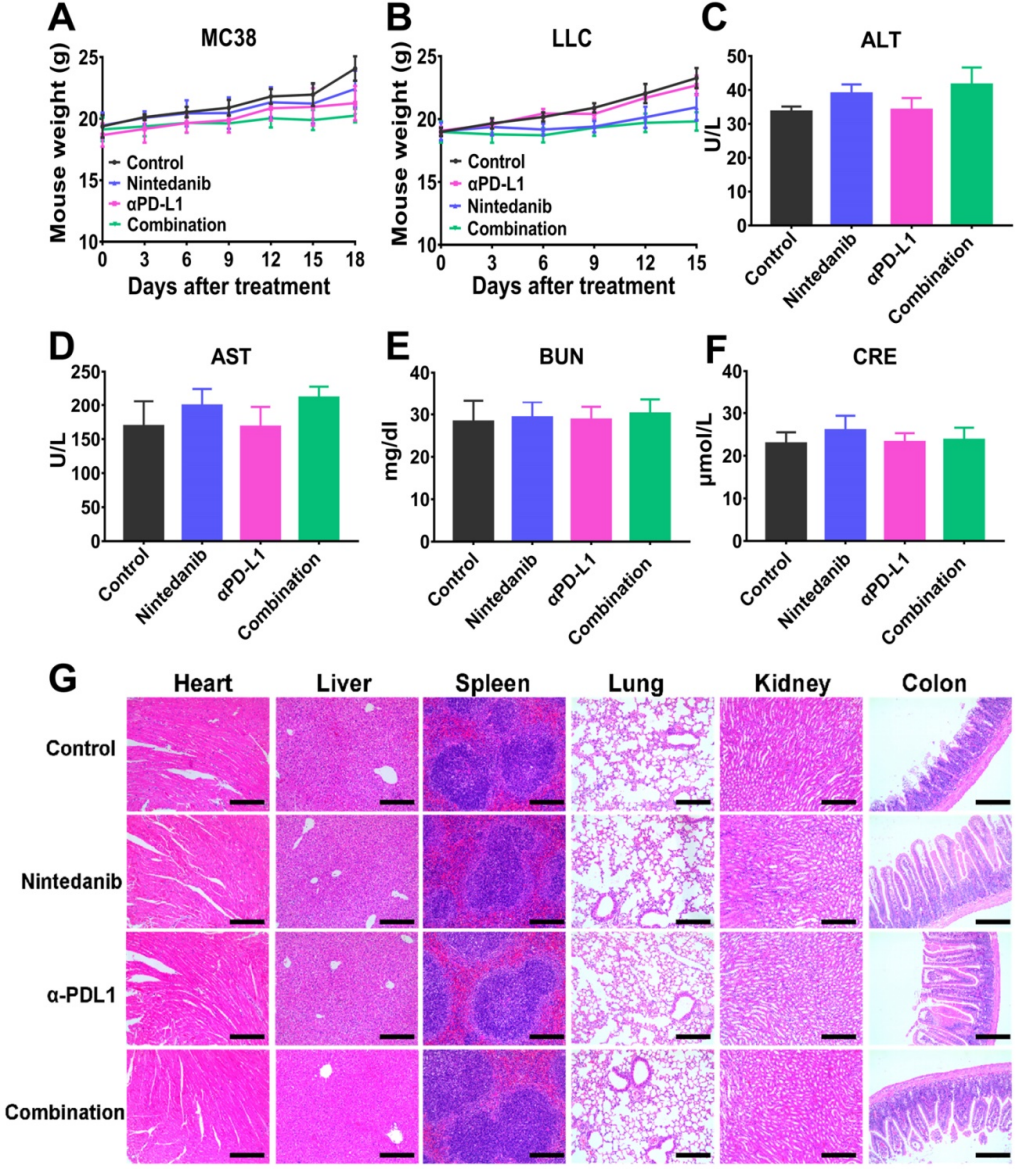
** The safety evaluation of different treatments.** Mouse weight in the **(A)** MC38 and **(B)** LLC models. The measurement of blood biochemistry parameters including **(C)** alanine aminotransferase (ALT), **(D)** aspartate aminotransferase (AST), **(E)** Blood urea nitrogen (BUN), and **(F)** Creatinine (CRE). **(G)** HE staining of major organs. The scale bar is 200 µm.

Although the previously presented results showed that nintedanib combined with αPD-L1 enhanced the efficacy of immunotherapy and antitumor effects, biosafety of this synergy therapy is still a primary concern in clinical practice. At the end of the animal experiment, mouse weights were recorded, and there was no significant weight loss in the treated mice compared to untreated mice, indicating that nintedanib was well tolerated *in vivo* (Figure 9A-B). The serum of the euthanized mice was also sampled for the measurement of blood biochemical parameters. As regular indicators of organ function, ALT, AST, BUN, and CRE were found to be within the normal range in this study (Figure 9C-F). Finally, there were no obvious pathological sites in HE staining of the main organs (Figure 9G). These results further demonstrate the safety in clinical application of this synergistic therapy.

## Discussion

**Figure 10:**
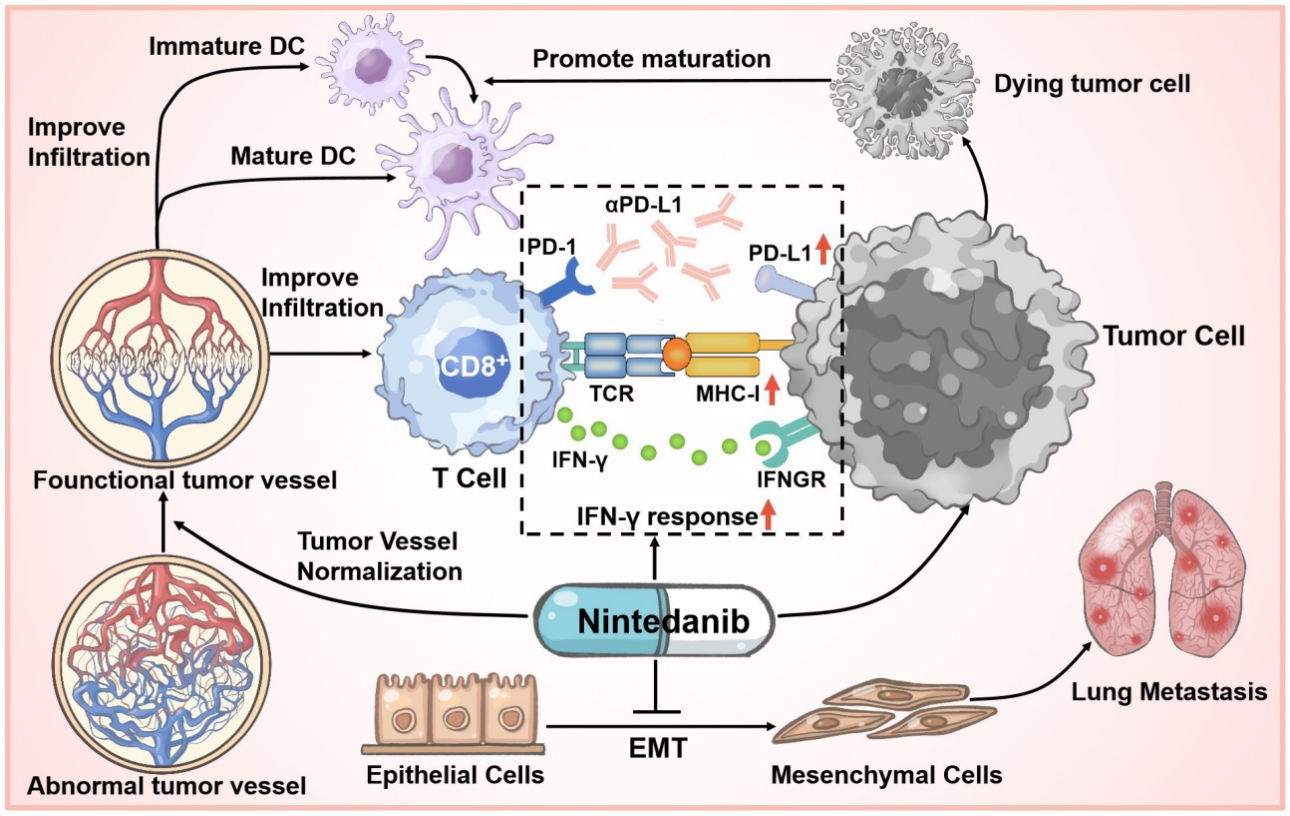
Schematic illustration of nintedanib effects in the tumor microenvironment (TME).

At present, although ICI therapy has been one of the optimal choices in partial tumor treatment cases, prolonging unprecedented survival in some patients, initial or acquired immunotherapy-related resistance in many cancers limits its effect and application 13, 18, 51. Based on extensive clinical research cases, only a small proportion of patients achieve a long-term and beneficial response, while immunotherapy resistance is an unfortunate experience for most patients. To solve this problem, multiagent cancer immunotherapy combination regimens have been widely recognized and explored, thus broadening the immunotherapy-responding population 52, 53. We are the first to report that nintedanib and αPD-L1 combination treatment not only increased the infiltration and activation of immune cells in tumor tissues through normalization of tumor blood vessels, but also upregulated the expression of PD-L1 and activated the MHC-I-mediated antigen processing and presentation process, thus improving the efficacy of immunotherapy and overcoming partial ICI resistance (Figure 10).

Currently, to explore effective antitumor mechanisms, various immune-related signaling pathways, such as the cGAS-STING pathway have been widely studied 54, 55. However, in our previous work, RNA-seq and pre-experiment results did not show that nintedanib therapy was related to the effects of these pathways. Despite this, it is undeniable that the combination of nintedanib and αPD-L1 exerted remarkable antitumor effects in multiple murine tumor-bearing models, and HE staining and TUNEL staining also showed this tendency. In a metastatic 4T1 tumor model, nintedanib treatment significantly reduced the metastatic nodules in the lung, which may be caused by inhibition of the EMT process 56, 57, reflecting its antimetastatic potential. Second, the distorted tumor blood vessel architecture and function normalization contributed to inhibition of tumor growth, recruitment of immune cells, improvement of tumor hypoxia, and transport of drugs 17, 19, 58. As an indicator of perfused functional vessels, the ratio of α-SMA/CD31 increased, while the number of tumor vessels was obviously reduced after nintedanib treatment, indicating tumor ECM vessel normalization 59. As for changes in the immune landscape, tumor tissue IHC and flow cytometry results reflected a substantial increase in the infiltration and activation of CD8^+^ T cells and promotion of DC cell maturation after combination treatment. This TME enhancement may be related to tumor vessel normalization and the induction of tumor cell apoptosis.

Moreover, the MC38 model tumor RNA-seq revealed that several immune responses were activated and enhanced after combination treatment, including improved IFN-γ response and MHC-I-mediated antigen processing and presentation. IFN-γ acting on tumor cells can upregulate MHC-I and PD-L1 expression 39, 60, 61. To simulate this process, different tumor cells received IFN-γ, nintedanib, or a combination treatment and the expression of MHC-I (β2M, H-2kb and H-2kd), TAP1 and PD-L1 was analyzed by western blot or flow cytometry. We found that combination treatment further upregulated MHC-I, TAP1 and PD-L1 expression compared to IFN-γ administered alone. Some studies have reported that STAT3 pathway activation may contribute to increase PD-L1, TAP1 and MHC-I levels 39, 62, 63. In addition, based on extensive research, the effect of nintedanib on the STAT3 pathway mainly depends on the concentration and cell responsiveness 64-66. Herein, our *in vitro* results demonstrated that a low concentration of nintedanib (1 μM) can promote STAT3 phosphorylation and downstream protein expression (PD-L1 and β2M) in different tumor cell lines (A549, MC38 and LLC) and gradually suppress these pathways at high doses (5 μM). This proved that nintedanib enhanced the IFN-γ response and the increased of MHC-I expression may be attributed to additional STAT3 pathway activation (Figure S7A-C). These results suggest that a low concentration of nintedanib may contribute to a better synergy when combined with ICIs. Regardless of whether nintedanib was used alone or in combination, its upregulation effect on PD-L1 has been verified in a variety of tumor cell lines. Recently, increasing evidence has shown that PD-L1 expression levels in tumor cells might affect the clinical response to anti-PD-1/PD-L1 therapies, and PD-L1 (+) patients have a better response rate to ICIs and better overall survival 39-44. The effect of nintedanib on tumor cells effectively increased the immune response of αPD-L1 and reshaped the TIME. Thus, nintedanib is an important complement to ICI resistance improvement.

On the other hand, one of major difficulties in tumor immunotherapy is complications in other organs 67. Among these, ILD limited ICI application and severely reduced the prognosis and survival of cancer patients 23, 24. Nintedanib, one of the most effective treatments for ILD, may be a valuable strategy for tumor patients complicated with ILD. Indeed, multiple clinical trials have shown that nintedanib combined with chemotherapy is well tolerated and has clinical benefits 68-70. Moreover, some patients who experienced disease progression after prior ICI therapy responded partially to the nintedanib combination strategy 71. Based on our work, nintedanib combined with αPD-L1 exerted notable antitumor and anti-pulmonary fibrosis effects and showed satisfactory safety in a IPF-tumor model. However, the αPD-L1 group had a higher mortality outcome than the control, which may have been caused by an aggravation in inflammation and fibrosis after ICI application 72, 73. There were no obvious differences in pulmonary fibrosis between αPD-L1 and the control group at the endpoint of experiment, possibly due to mice with severe pulmonary fibrosis dying earlier (Figure 8C and 8F-H). These encouraging results show that nintedanib has the potential to alleviate pulmonary fibrosis, increase the efficacy of immunotherapy and overcome ICI resistance and side effects. Our results provide convincing evidence to support the advancement of this synergistic therapy to clinical trials. To translate our results into practical clinical applications, further clinical evaluation is necessary to determine the efficacy and safety of nintedanib and αPD-L1 combination therapy. Therefore, our team is preparing to launch a Phase II clinical trial of nintedanib combined with αPD-L1.

However, there are some limitations to this study. Herein, we suggest that nintedanib and αPD-L1 combination treatment improves the immune response by enhancing the tumor cell IFN-γ response and increasing PD-L1 and MHC-I levels. Further investigation into specific targets, regulatory relationships that influence these pathways, and studies on whether nintedanib is suitable for other combination regimens, such as radiotherapy, are necessary. Due to the upregulation of PD-L1 and MHC-I 74, radiotherapy with nintedanib and αPD-L1 synergy therapy may be a valuable combination worthy of further study. Moreover, the efficacy of the combined treatment was weaker against the CT26 cell line, which is a pMMR (MSS), responding poorly to immunotherapy compared to the efficacy against other tumor cells 75, 76. Unfortunately, these results imply that patients with MSS tumors, in clinical settings, may only partially benefit from this synergistic therapy. Despite this, it is undeniable that nintedanib and αPD-L1 combination treatment exerts excellent antitumor effects and overcomes partial ICI therapy resistance in multiple cancers.

## Conclusions

We found, with a series of *in vivo* and* in vitro* experiments, that nintedanib and αPD-L1 combination treatment exhibited significant antitumor and antimetastatic effects. Further investigation of these effects revealed that nintedanib could promote tumor vascular normalization and increase T cell and DC infiltration and activation while reshaping the TIME (enhanced IFN-γ responses and MHC-I-mediated antigen presentation). Moreover, as the upregulation of PD-L1 expression increased the tumor cell response to αPD-L1, nintedanib combined with αPD-L1 further exhibited its immune activation effects; thus, enhancing the efficacy of ICI therapy. Based on these encouraging results, nintedanib and αPD-L1 synergy therapy may provide a potential strategy for the treatment of tumor patients with immunotherapy resistance and ILD.

## Supplementary Material

Supplementary figures and tables.

## Abbreviations

ALT: alanine aminotransferase
ANOVA: analysis of variance
AST: aspartate aminotransferase
BUN: blood urea nitrogen
CRE: creatinine
DCs: dendritic cells
DEGs: differentially expressed genes
EMT: epithelial-mesenchymal transition
ECM: extra cellular matrix
GO: gene ontology
GSEA: gene set enrichment analysis
HE: hematoxylin-eosin
HUVECs: human umbilical vein endothelial cells
ICIs: immune checkpoint inhibitors
ILD: interstitial lung diseases
IF: immunofluorescence
IHC: immunohistochemistry
IFN-γ: interferon-gamma
IPF: idiopathic pulmonary fibrosis
KEGG: kyoto encyclopedia of genes and genomes
MHC-I: major histocompatibility complex class I
MDSCs: myeloid-derived suppressor cells
NSCLC: non-small cell lung cancer
PD-1: programmed cell death protein 1
PD-L1: programmed death-ligand 1
RT-PCR: Real-time PCR
rMuIFN-γ: recombinant mouse interferon-gamma
rHuIFN-γ: recombinant human interferon-gamma
RNA-seq: RNA sequencing
TAP: Transporter associated with antigen processing
TIME: Tumor immune microenvironment
TME: Tumor microenvironment
TAMs: tumor-associated macrophages
TDLN: tumor-draining lymph node
β2M: beta-2-microglobulin
