# Supplementary material for: Nintedanib enhances the efficacy of PD-L1 blockade by upregulating MHC-I and PD-L1 expression in tumor cells
Source: Theranostics. 2022 Jan 1;12(2):747–66. doi: 10.7150/thno.65828 (PMC8692903; doi:10.7150/thno.65828)
Supplement: Supplementary file 1 — Supplementary figures and tables. [file thnov12p0747s1.pdf]

Supplementary Material

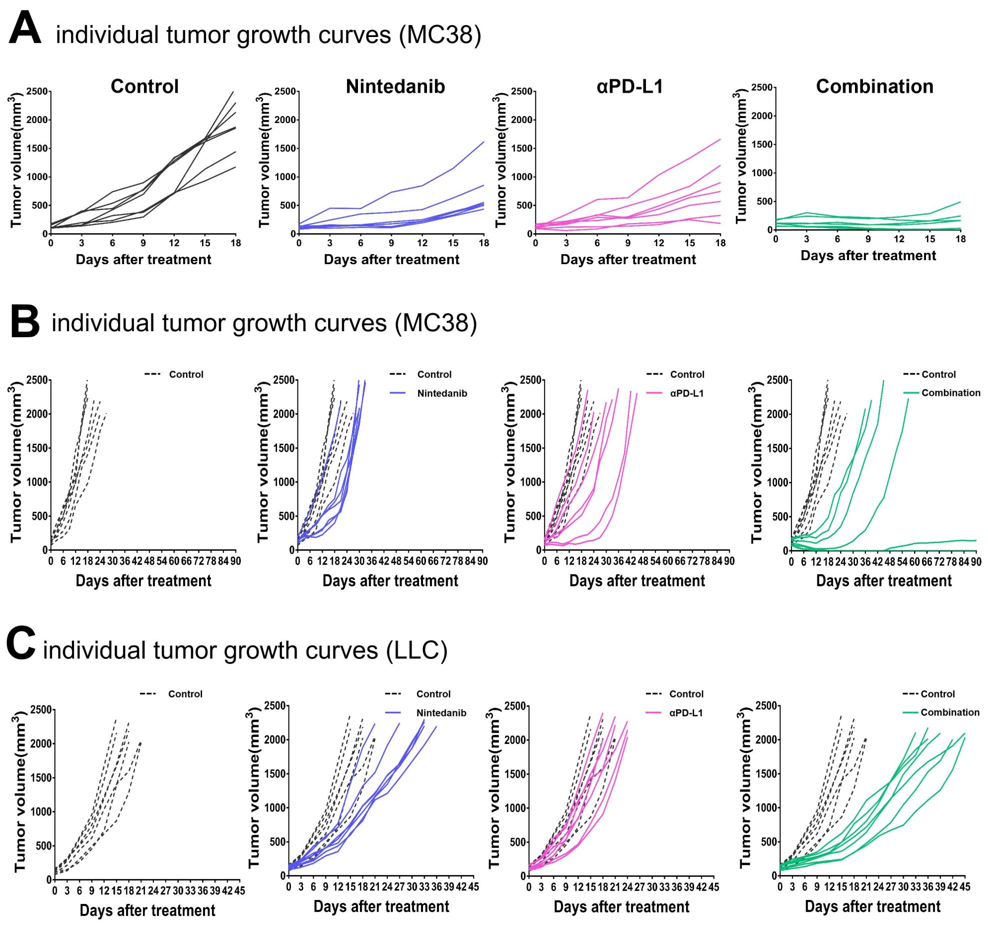

Figure S1. Individual tumor volume curves of different mouse models. Individual tumor volume curves for the (A) MC38 tumor inhibition experiment, (B) MC38 and (C) LLC survival experiment.

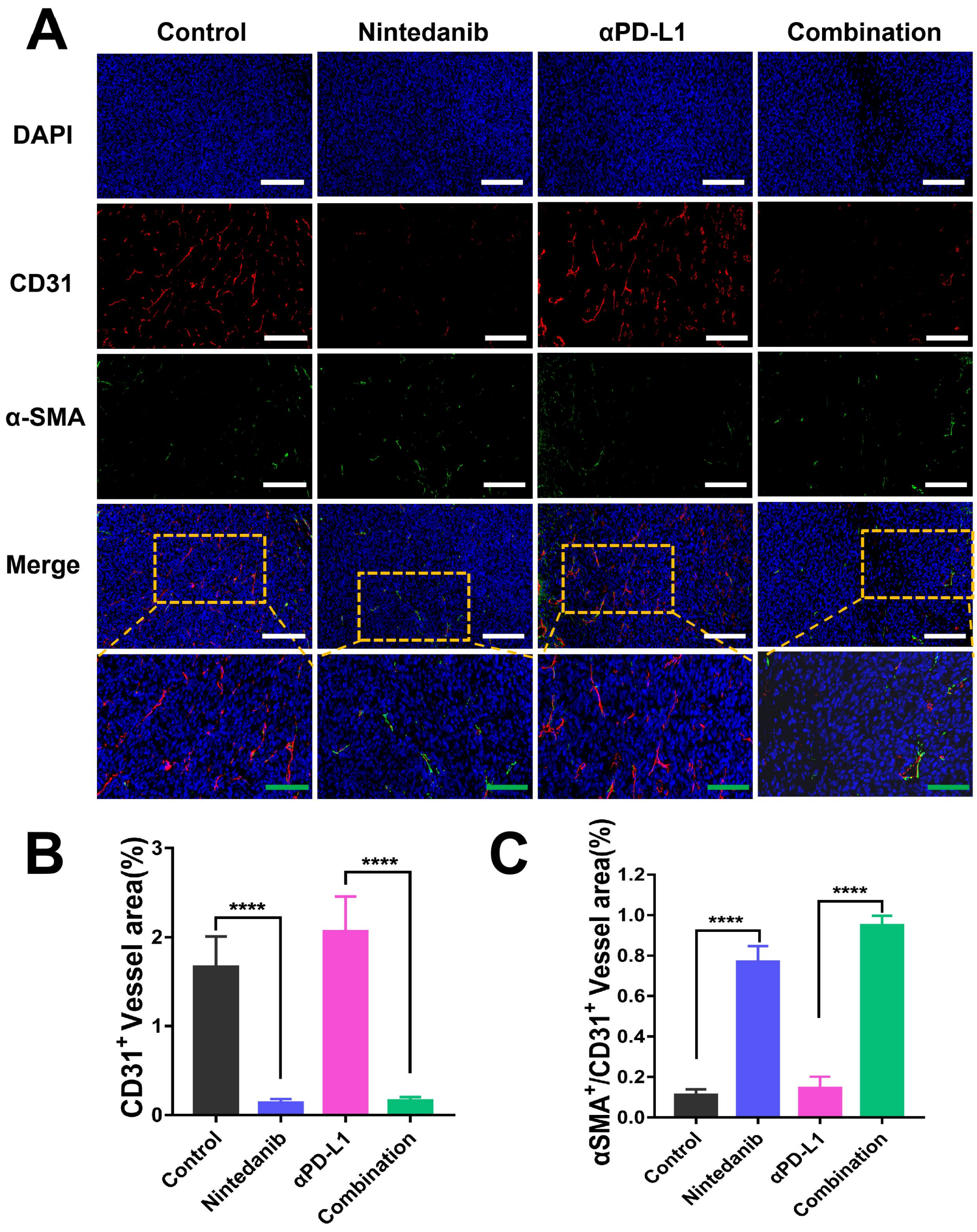

Figure S2. Immunofluorescence (IF) analysis of vessel normalization of MC38 tumor. (A) IF images of CD31 and α-SMA in MC38 tumor tissue. The quantitative analysis of (B) $CD31^+$ vessel area and (C) $α\text{-}SMA^+/CD31^+$ vessel area. The green scale bar is 100 μm and the white scale bar is 200 μm.

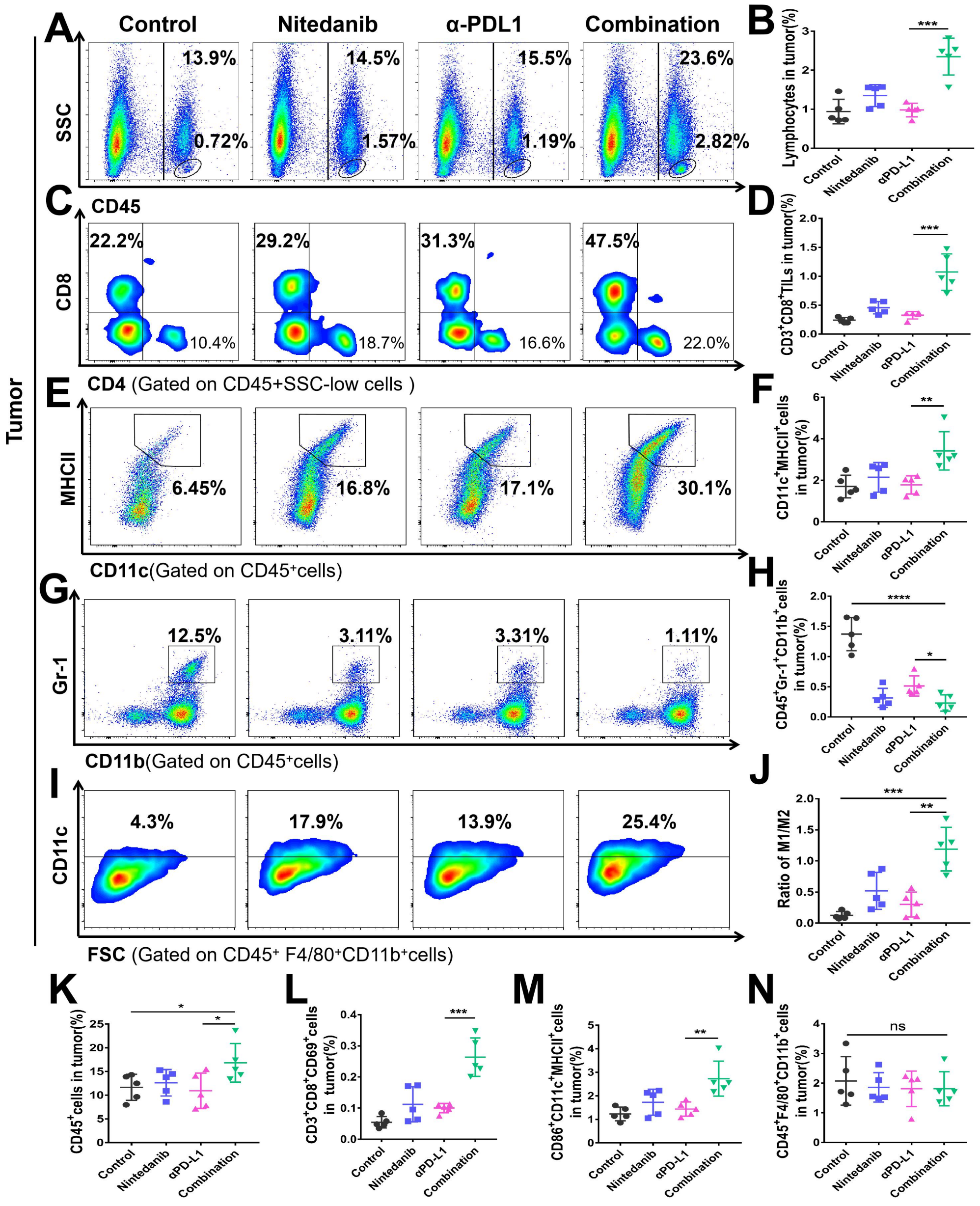

Figure S3. Flow cytometry analysis of the tumor immune microenvironment (TIME) in LLC tumor tissue. Representative images of flow cytometry of (A) lymphocytes, (C) $CD8^{+}$ T cells (gated on $CD45^{+}$ cells + SSC-low cells), (E) $CD11c^{+}MHCII^{+}$ cells (gated on $CD45^{+}$ cells), (G) $Gr\text{-}1^{+}CD11b^{+}$ cells (gated on $CD45^{+}$ cells), and (I) $CD11c^{+}$ cells (gated on $CD45^{+}F4/80^{+}CD11b^{+}$ cells). Quantitative analysis of the proportion of (B) lymphocytes, (D) $CD3^{+}CD8^{+}$ TILs, (F) $CD11c^{+}MHCII^{+}$ cells, (H) $CD45^{+}$ $Gr\text{-}1^{+}$ $CD11b^{+}$ cells, (J)M1/M2, (K) $CD45^{+}$ cells, (L) $CD3^{+}CD8^{+}CD69^{+}$ cells, (M) $CD86^{+}CD11c^{+}MHCII^{+}$ cells, and (N) $CD45^{+}F4/80^{+}CD11b^{+}$ cells in the tumor.

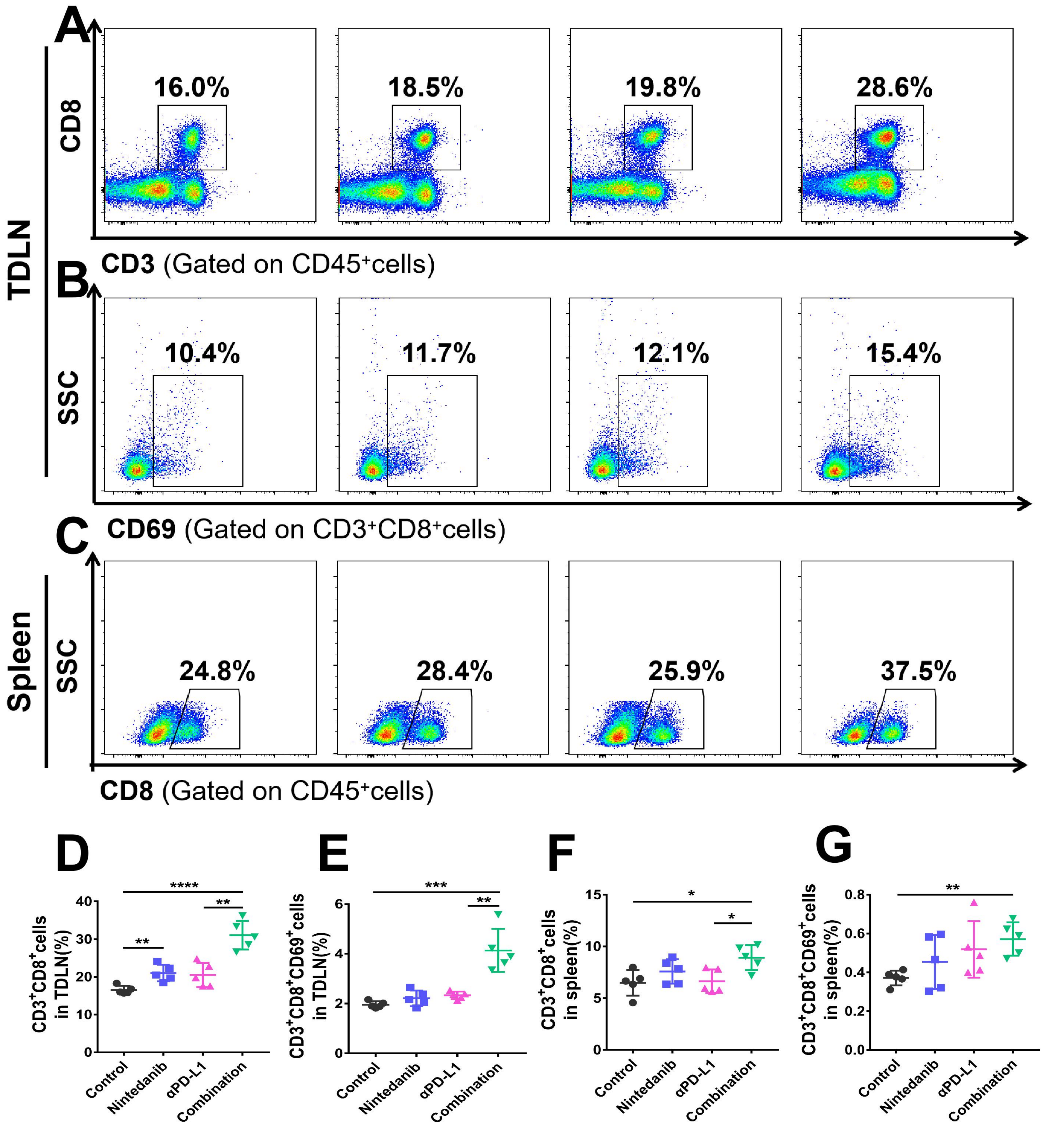

Figure S4. Flow cytometry analysis of the immune microenvironment in LLC model TDLN and spleen. Representative images of flow cytometry of (A) $CD3^+CD8^+$ T cells (gated on $CD45^+$ cells) and (B) $CD69^+$ T cells (gated on $CD3^+CD8^+$ cells) in the TDLN, and (C) $CD8^+$ T cells (gated on $CD45^+$ cells) in the spleen. Quantitative analysis of the proportion of (D) $CD3^+CD8^+$ cells and (E) $CD3^+CD8^+CD69^+$ cells in the TDLN, and (F) $CD3^+CD8^+$ cells and (G) $CD3^+CD8^+CD69^+$ cells in the spleen.

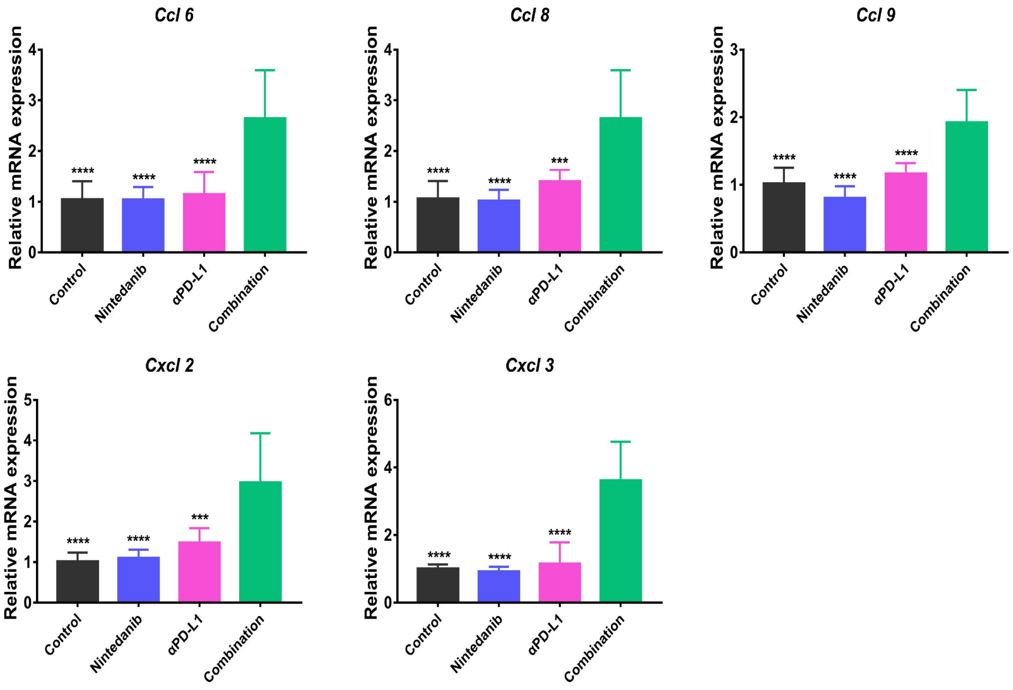

Figure S5. Real-time PCR (RT-PCR) validation of selected genes identified by RNA-seq.

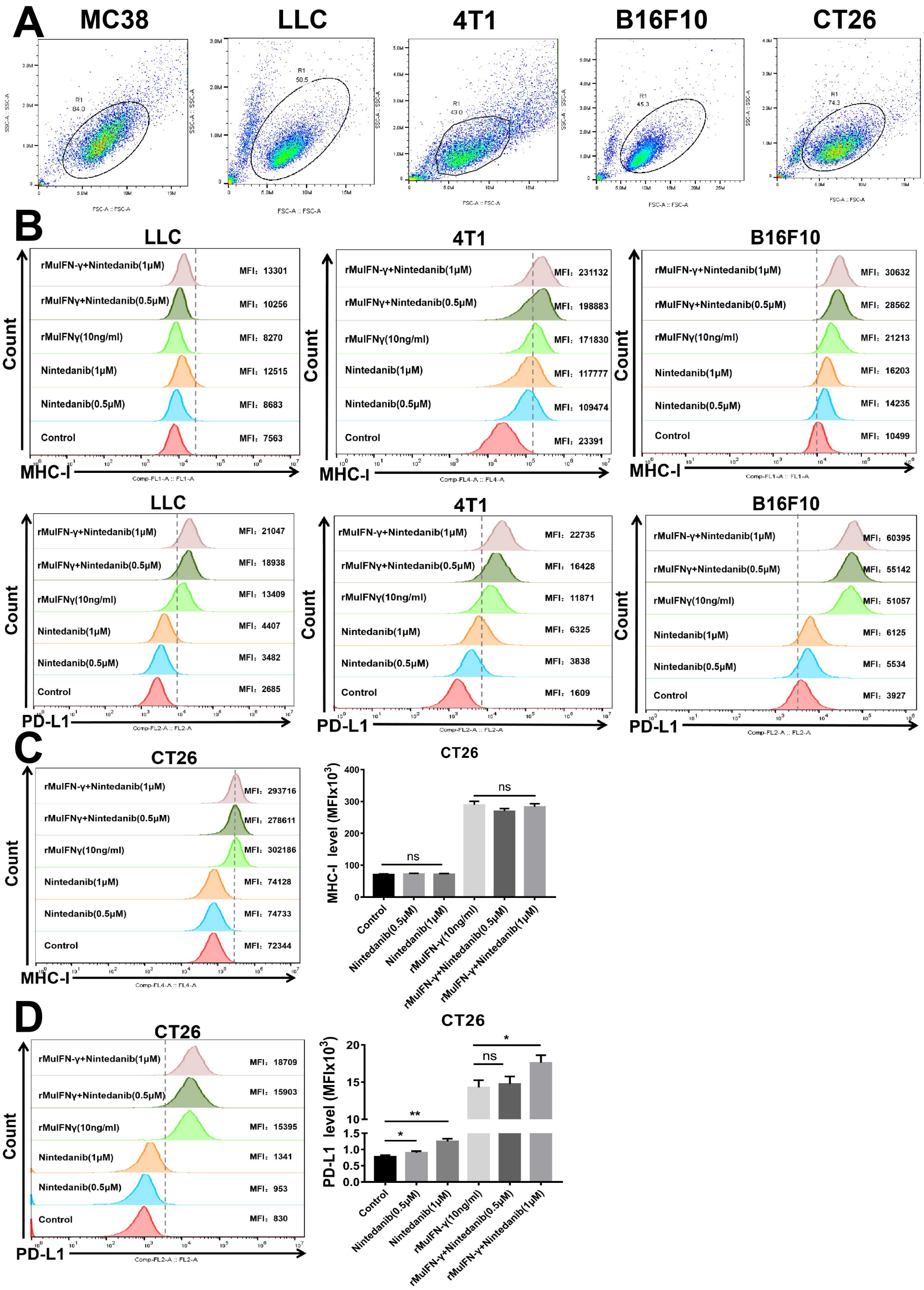

Figure S6. Detection of MHC-I and PD-L1 expression in a variety of tumor cell lines. (A) Schematic diagrams of gating on tumor cells. (B) Representative images of MHC-I and PD-L1 levels in different tumor cells. Representative images and quantification of (C) MHC-I and (D) PD-L1 levels in CT26 cells.

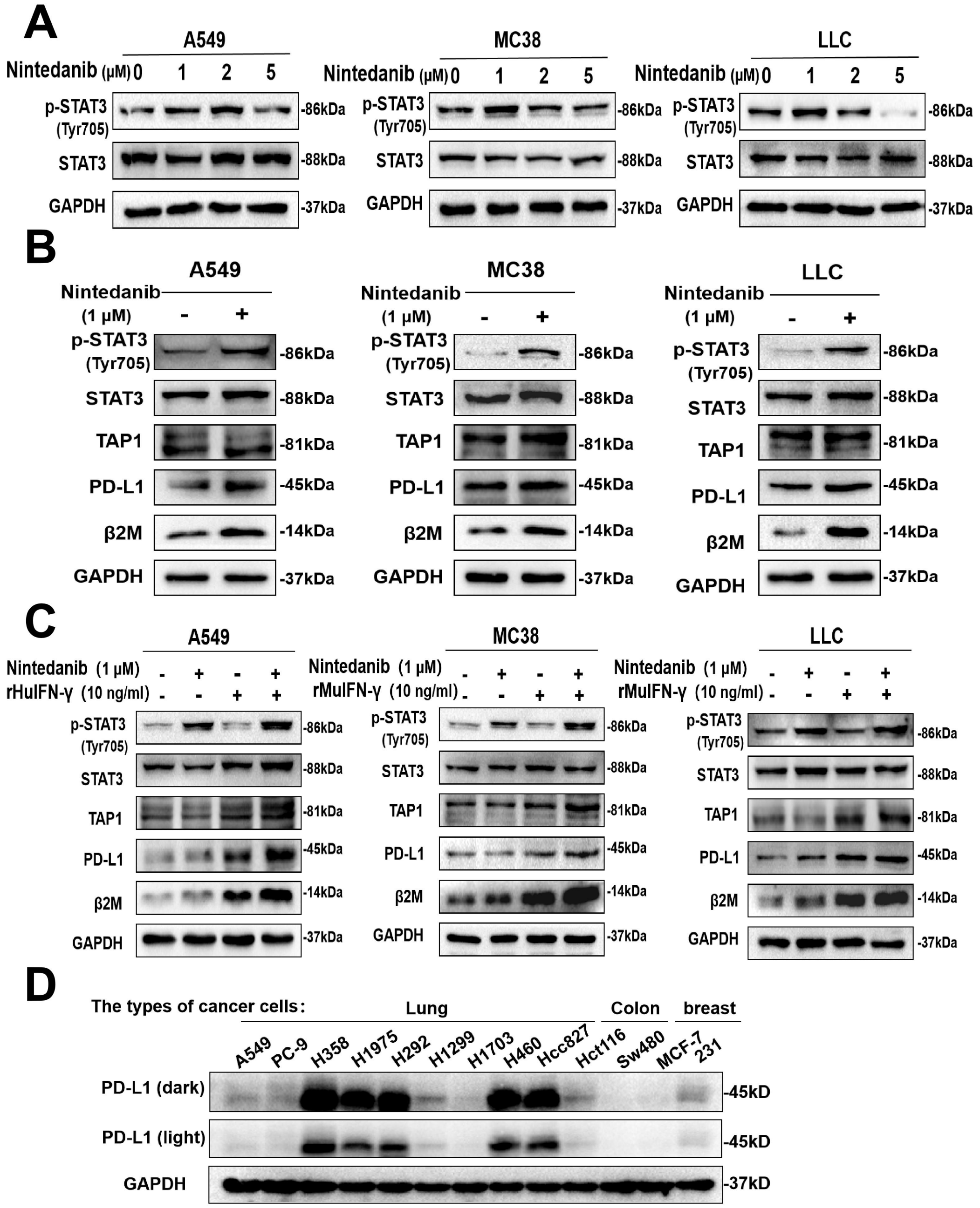

Figure S7. The effect of nintedanib on different tumor cells (A549, MC38 and LLC). (A) Western blotting assays of p-STAT3/STAT3 under different concentrations of nintedanib treatment for 24 h. Western blotting assays of p-STAT3/STAT3, TAP1, PD-L1 and β2M after (B) 1 μM nintedanib and (C) combination treatment for 24 h. (D) The basal levels of PD-L1 in different tumor cells.

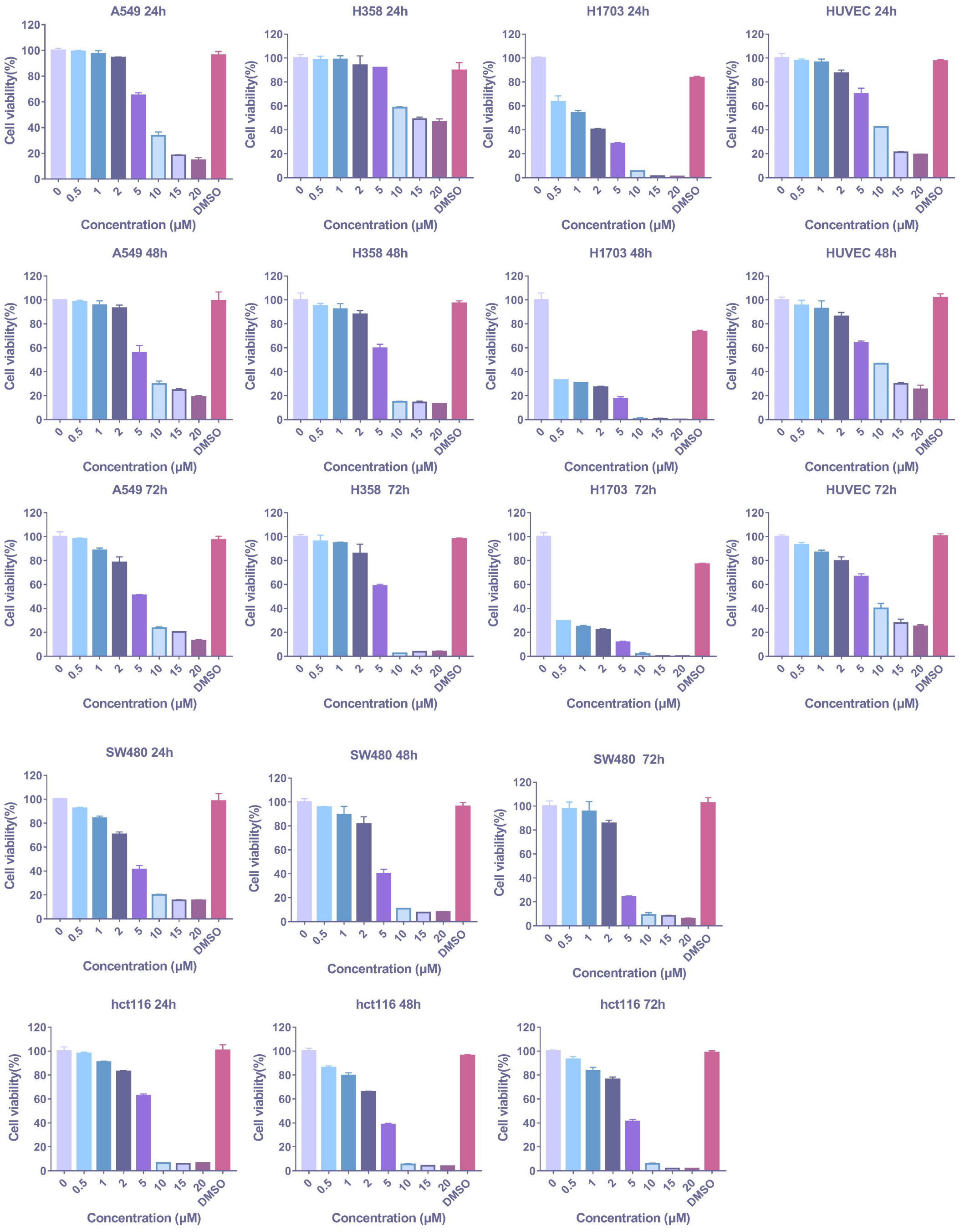

Figure S8. Various human tumor cells and HUVEC treated with different concentrations of nintedanib for 24, 48, or 72 h and detection of cytotoxicity using CCK-8 assays.

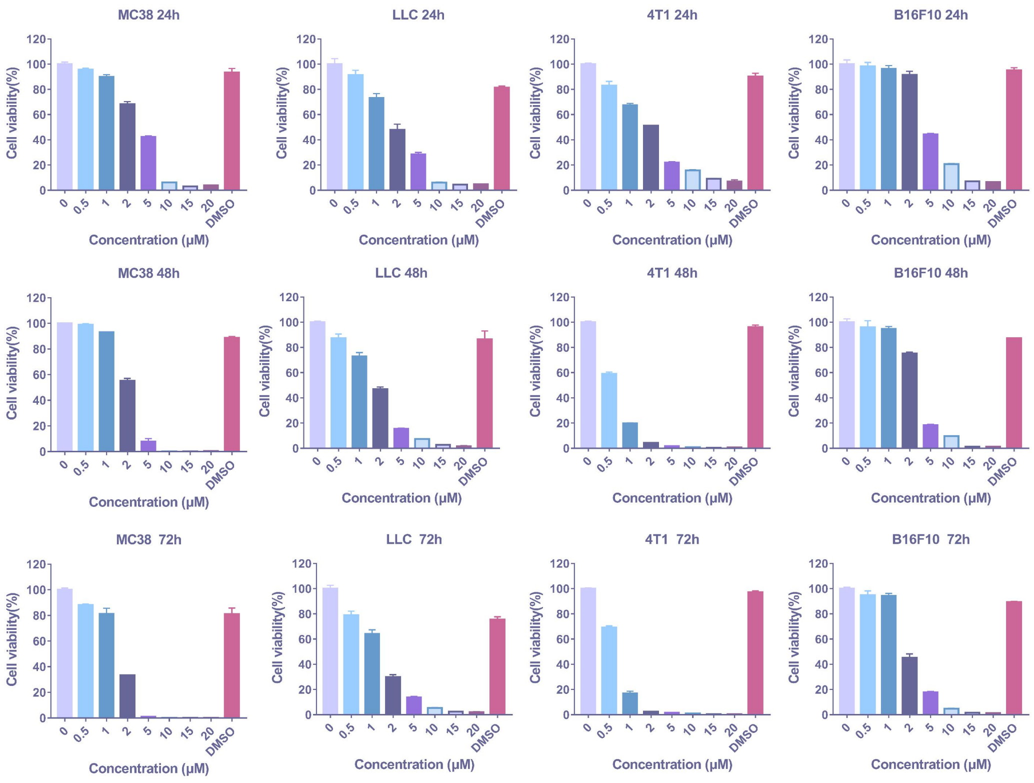

Figure S9. Various mouse tumor cells treated with different concentrations of nintedanib for 24, 48, or 72 h and detection of cytotoxicity using CCK-8.

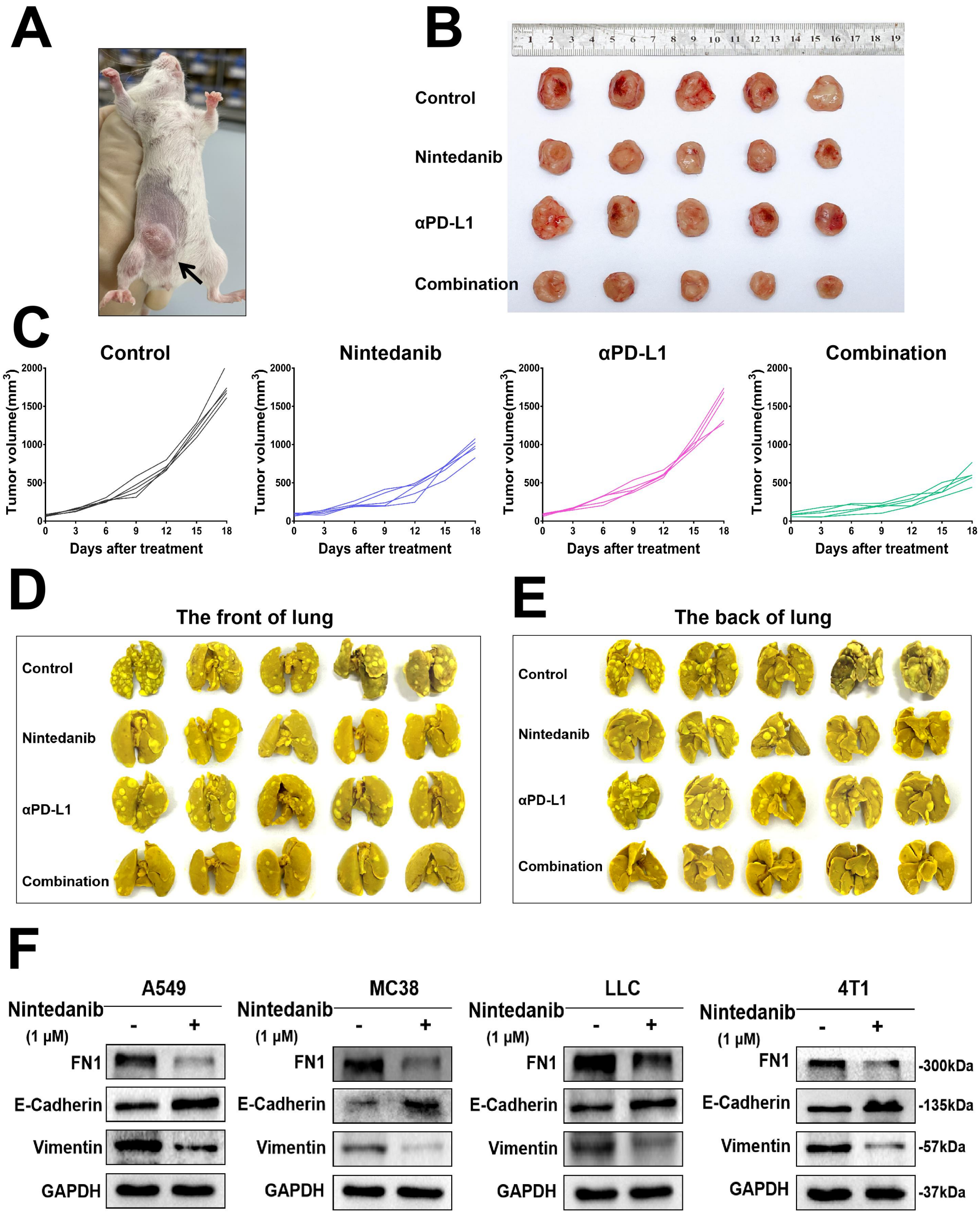

Figure S10. Tumor growth and metastasis in the lungs of the 4T1 model. (A) Schematic diagram of the 4T1 tumor-bearing model (black arrow is 4T1 tumor). (B) Images and (C) individual tumor volume curves of 4T1 tumors. The (D) front and (E) back view of individual lung tissues. (F) Western blotting assays of epithelial-mesenchymal transition (EMT) related indicators in different tumor cells after nintedanib treatment *in vitro* for 24 h.

Table S1. Union list of the top 100 immune-related GO terms for the DEGs from the combination vs. control groups, αPD-L1 vs. control groups, and nintedanib vs. control groups.

GOID	Description	GeneRatio	padj
GO:0050778	positive regulation of immune response	66/683	2.40E-14
GO:0001819	positive regulation of cytokine production	56/683	9.28E-14
GO:0034341	response to interferon-gamma	31/683	1.03E-13
GO:0002474	antigen processing and presentation of peptide antigen via MHC class I	18/683	3.83E-13
GO:0002697	regulation of immune effector process	50/683	7.04E-13
GO:0071346	cellular response to interferon-gamma	27/683	1.15E-12
GO:0097530	granulocyte migration	29/683	1.49E-12
GO:1990266	neutrophil migration	26/683	2.54E-12
GO:0019882	antigen processing and presentation	25/683	2.76E-12
GO:0048002	antigen processing and presentation of peptide antigen	20/683	5.90E-12
GO:0071621	granulocyte chemotaxis	26/683	6.33E-12
GO:0030593	neutrophil chemotaxis	23/683	1.33E-11
GO:0030595	leukocyte chemotaxis	34/683	3.30E-11
GO:0050900	leukocyte migration	43/683	4.44E-11
GO:0002694	regulation of leukocyte activation	54/683	3.97E-10
GO:1903555	regulation of tumor necrosis factor superfamily cytokine production	27/683	5.31E-10
GO:0002250	adaptive immune response	45/683	6.55E-10
GO:0071706	tumor necrosis factor superfamily cytokine production	27/683	8.68E-10
GO:0032635	interleukin-6 production	26/683	9.69E-10
GO:0002703	regulation of leukocyte mediated immunity	32/683	1.07E-09
GO:0002460	adaptive immune response based on somatic recombination of immune receptors built from immunoglobulin superfamily domains	36/683	1.09E-09
GO:0002443	leukocyte mediated immunity	41/683	1.29E-09
GO:1903557	positive regulation of tumor necrosis factor superfamily cytokine production	21/683	1.34E-09
GO:0032675	regulation of interleukin-6 production	25/683	1.63E-09
GO:0002483	antigen processing and presentation of endogenous peptide antigen	9/683	2.02E-09
GO:0045088	regulation of innate immune response	35/683	4.57E-09
GO:0032760	positive regulation of tumor necrosis factor production	20/683	7.49E-09
GO:0032680	regulation of tumor necrosis factor production	25/683	9.15E-09
GO:0032640	tumor necrosis factor production	25/683	1.23E-08
GO:0002449	lymphocyte mediated immunity	33/683	1.71E-08
GO:0019883	antigen processing and presentation of endogenous antigen	9/683	2.00E-08
GO:0019221	cytokine-mediated signaling pathway	41/683	2.02E-08
GO:0035456	response to interferon-beta	15/683	3.98E-08
GO:0002696	positive regulation of leukocyte activation	36/683	1.07E-07

GO:0002253	activation of immune response	39/683	2.05E-07
GO:0035458	cellular response to interferon-beta	13/683	2.17E-07
GO:0042110	T cell activation	46/683	3.58E-07
GO:0051249	regulation of lymphocyte activation	42/683	3.64E-07
GO:0070661	leukocyte proliferation	35/683	4.03E-07
GO:0002706	regulation of lymphocyte mediated immunity	23/683	8.24E-07
GO:0070098	chemokine-mediated signaling pathway	13/683	1.29E-06
GO:0002699	positive regulation of immune effector process	27/683	1.40E-06
GO:0007159	leukocyte cell-cell adhesion	34/683	1.81E-06
GO:0002822	regulation of adaptive immune response based on somatic recombination of immune receptors built from immunoglobulin superfamily domains	22/683	2.01E-06
GO:0002819	regulation of adaptive immune response	23/683	2.07E-06
GO:0051251	positive regulation of lymphocyte activation	30/683	2.43E-06
GO:0046651	lymphocyte proliferation	32/683	3.70E-06
GO:0001909	leukocyte mediated cytotoxicity	18/683	4.13E-06
GO:0002521	leukocyte differentiation	47/683	4.28E-06
GO:0070665	positive regulation of leukocyte proliferation	21/683	6.19E-06
GO:0001910	regulation of leukocyte mediated cytotoxicity	15/683	6.65E-06
GO:0045089	positive regulation of innate immune response	26/683	6.67E-06
GO:1902107	positive regulation of leukocyte differentiation	22/683	7.87E-06
GO:0002764	immune response-regulating signaling pathway	32/683	1.24E-05
GO:0032946	positive regulation of mononuclear cell proliferation	20/683	1.46E-05
GO:0002685	regulation of leukocyte migration	24/683	1.46E-05
GO:0002705	positive regulation of leukocyte mediated immunity	19/683	1.55E-05
GO:0002688	regulation of leukocyte chemotaxis	18/683	1.59E-05
GO:0050900	leukocyte migration	19/357	5.71E-03
GO:0060326	cell chemotaxis	15/357	3.87E-02
GO:0071346	cellular response to interferon-gamma	9/357	4.07E-02
GO:0030593	neutrophil chemotaxis	11/71	3.96E-11
GO:1990266	neutrophil migration	11/71	1.67E-10
GO:0071621	granulocyte chemotaxis	11/71	1.78E-10
GO:0030595	leukocyte chemotaxis	13/71	2.94E-10
GO:0060326	cell chemotaxis	14/71	5.34E-10
GO:0097530	granulocyte migration	11/71	5.34E-10
GO:0006935	chemotaxis	17/71	4.00E-09
GO:0050900	leukocyte migration	13/71	3.65E-08
GO:0002250	adaptive immune response	13/71	2.37E-07
GO:0002460	adaptive immune response based on somatic recombination of immune receptors built from immunoglobulin superfamily domains	10/71	5.99E-06
GO:0090022	regulation of neutrophil chemotaxis	5/71	3.41E-05
GO:0002694	regulation of leukocyte activation	12/71	4.59E-05
GO:0006909	phagocytosis	8/71	6.06E-05
GO:0050778	positive regulation of immune response	12/71	8.60E-05

GO:1902622	regulation of neutrophil migration	5/71	8.60E-05
GO:0002757	immune response-activating signal transduction	9/71	1.68E-04
GO:0071622	regulation of granulocyte chemotaxis	5/71	1.68E-04
GO:0002696	positive regulation of leukocyte activation	9/71	2.00E-04
GO:0050718	positive regulation of interleukin-1 beta secretion	4/71	2.08E-04
GO:0002764	immune response-regulating signaling pathway	9/71	2.13E-04
GO:0050716	positive regulation of interleukin-1 secretion	4/71	2.21E-04
GO:0050766	positive regulation of phagocytosis	5/71	2.21E-04
GO:0051249	regulation of lymphocyte activation	10/71	2.46E-04
GO:0002429	immune response-activating cell surface receptor signaling pathway	7/71	2.69E-04
GO:0090023	positive regulation of neutrophil chemotaxis	4/71	3.07E-04
GO:0002688	regulation of leukocyte chemotaxis	6/71	3.07E-04
GO:0050706	regulation of interleukin-1 beta secretion	4/71	3.33E-04
GO:0051251	positive regulation of lymphocyte activation	8/71	3.41E-04
GO:0002768	immune response-regulating cell surface receptor signaling pathway	7/71	3.42E-04
GO:0071624	positive regulation of granulocyte chemotaxis	4/71	3.42E-04
GO:0002253	activation of immune response	9/71	4.23E-04
GO:0032731	positive regulation of interleukin-1 beta production	4/71	4.23E-04
GO:0002685	regulation of leukocyte migration	7/71	4.26E-04
GO:0034341	response to interferon-gamma	6/71	4.78E-04
GO:0050704	regulation of interleukin-1 secretion	4/71	4.91E-04
GO:0050702	interleukin-1 beta secretion	4/71	5.28E-04
GO:1902624	positive regulation of neutrophil migration	4/71	5.28E-04
GO:0042110	T cell activation	10/71	5.52E-04
GO:0050920	regulation of chemotaxis	7/71	5.93E-04
GO:0050921	positive regulation of chemotaxis	6/71	5.93E-04
GO:0046651	lymphocyte proliferation	8/71	5.93E-04
GO:0032732	positive regulation of interleukin-1 production	4/71	5.93E-04
GO:0032680	regulation of tumor necrosis factor production	6/71	5.93E-04
GO:0032943	mononuclear cell proliferation	8/71	5.93E-04
GO:0032640	tumor necrosis factor production	6/71	6.13E-04
GO:1903555	regulation of tumor necrosis factor superfamily cytokine production	6/71	6.13E-04
GO:0050764	regulation of phagocytosis	5/71	6.38E-04
GO:0050871	positive regulation of B cell activation	5/71	6.38E-04
GO:0071706	tumor necrosis factor superfamily cytokine production	6/71	6.60E-04
GO:0032760	positive regulation of tumor necrosis factor production	5/71	6.96E-04
GO:0070661	leukocyte proliferation	8/71	6.97E-04
GO:1903557	positive regulation of tumor necrosis factor superfamily cytokine production	5/71	7.14E-04
GO:0050701	interleukin-1 secretion	4/71	7.33E-04
GO:0002690	positive regulation of leukocyte chemotaxis	5/71	7.33E-04
GO:0007159	leukocyte cell-cell adhesion	8/71	7.40E-04
GO:0030890	positive regulation of B cell proliferation	4/71	7.76E-04
GO:0001819	positive regulation of cytokine production	9/71	8.41E-04

GO:0002449	lymphocyte mediated immunity	7/71	1.06E-03
GO:0002521	leukocyte differentiation	10/71	1.07E-03
GO:0002285	lymphocyte activation involved in immune response	6/71	1.11E-03
GO:0002824	positive regulation of adaptive immune response based on somatic recombination of immune receptors built from immunoglobulin superfamily domains	5/71	1.12E-03
GO:0006911	phagocytosis, engulfment	4/71	1.19E-03
GO:0032651	regulation of interleukin-1 beta production	4/71	1.28E-03
GO:0002821	positive regulation of adaptive immune response	5/71	1.43E-03
GO:0071346	cellular response to interferon-gamma	5/71	1.43E-03
GO:0001776	leukocyte homeostasis	5/71	1.71E-03
GO:0046635	positive regulation of alpha-beta T cell activation	4/71	1.89E-03
GO:0030098	lymphocyte differentiation	8/71	1.89E-03
GO:0043372	positive regulation of CD4-positive, alpha-beta T cell differentiation	3/71	2.52E-03
GO:0032611	interleukin-1 beta production	4/71	2.52E-03
GO:0032652	regulation of interleukin-1 production	4/71	2.83E-03
GO:0030888	regulation of B cell proliferation	4/71	2.98E-03
GO:0050864	regulation of B cell activation	5/71	3.02E-03
GO:0032675	regulation of interleukin-6 production	5/71	3.07E-03
GO:0042098	T cell proliferation	6/71	3.12E-03
GO:0042116	macrophage activation	4/71	3.49E-03
GO:0032635	interleukin-6 production	5/71	3.58E-03
GO:0050671	positive regulation of lymphocyte proliferation	5/71	3.58E-03
GO:2000516	positive regulation of CD4-positive, alpha-beta T cell activation	3/71	3.58E-03
GO:0032946	positive regulation of mononuclear cell proliferation	5/71	3.80E-03
GO:0002687	positive regulation of leukocyte migration	5/71	4.14E-03
GO:0070665	positive regulation of leukocyte proliferation	5/71	4.24E-03
GO:0050670	regulation of lymphocyte proliferation	6/71	4.26E-03
GO:0002443	leukocyte mediated immunity	7/71	4.39E-03
GO:0032944	regulation of mononuclear cell proliferation	6/71	4.39E-03

Table S2. List of the top 10 significantly enriched immune-related GO terms (combination vs. control groups and combination vs. αPD-L1 groups).

Combination vs Control			
GOID	Description	GeneRatio	padj
GO:0050778	positive regulation of immune response	66/683	2.40E-14
GO:0001819	positive regulation of cytokine production	56/683	9.28E-14
GO:0034341	response to interferon-gamma	31/683	1.03E-13
GO:0002474	antigen processing and presentation of peptide antigen via MHC class I	18/683	3.83E-13
GO:0002697	regulation of immune effector process	50/683	7.04E-13
GO:0097530	granulocyte migration	29/683	1.49E-12
GO:0060326	cell chemotaxis	41/683	4.60E-12
GO:0031349	positive regulation of defense response	45/683	5.97E-12
GO:0030593	neutrophil chemotaxis	23/683	1.33E-11
GO:0030595	leukocyte chemotaxis	34/683	3.30E-11

Combination vs αPD-L1			
GOID	Description	GeneRatio	padj
GO:0035456	response to interferon-beta	13/172	4.48203E-12
GO:0098542	defense response to other organism	22/172	7.37877E-08
GO:0034341	response to interferon-gamma	13/172	2.42527E-07
GO:0002474	antigen processing and presentation of peptide antigen via MHC class I	8/172	7.17085E-07
GO:0052547	regulation of peptidase activity	18/172	4.70726E-06
GO:0019221	cytokine-mediated signaling pathway	17/172	1.28654E-05
GO:0070098	chemokine-mediated signaling pathway	7/172	5.23749E-05
GO:0006959	humoral immune response	10/172	6.3301E-05
GO:0002697	regulation of immune effector process	15/172	0.000207045
GO:0002449	lymphocyte mediated immunity	12/172	0.000323608

Table S3. List of enriched signature genes of the IFN-γ response heat map.

gene_name	Control_Mean	Nintedanib_Mean	PD_L1_Mean	Combination_Mean
C1ra	-0.902484374	-0.086503448	-0.441187059	1.451800744
Zbp1	-1.042175738	0.189987078	-0.328361954	1.133053845
Apol6	-0.760255649	-0.31399258	-0.328302905	1.481049279
AC168977.1	-0.405240103	-0.01012377	-0.787854563	1.205749378
AC133103.1	-1.047997003	0.510245622	-0.571098436	0.98128841
Apol9a	-0.306893593	-0.042330034	-0.73804231	1.097848445
H2-T22	-0.805996553	-0.065049204	-0.428396582	1.315704641
H2-T23	-0.635567103	-0.288505978	-0.375467674	1.37166725
Psmb9	-0.694521069	-0.062797968	-0.497838087	1.270856615
Tap1	-0.500941282	-0.393740957	-0.416591586	1.409709064
Bst2	-0.52444032	-0.138235268	-0.448201788	1.145436193
Mx2	-0.318862773	0.014705432	-0.667264422	0.967745405
Ccl8	-0.81956612	-0.471716384	-0.052700638	1.461912238
Ccl2	-0.491391404	-0.117103794	-0.514556232	1.152327378
Xaf1	-0.959271674	0.106584953	-0.223832577	1.04987306
Ifi27l2a	-0.452940402	0.139426252	-0.723056307	1.001713895
Ifitm3	-0.689908139	0.044190217	-0.585159994	1.219830361
Cdkn1a	-0.451283155	-0.20494524	-0.549673281	1.257137986
Dhx58	-0.576927393	0.03833996	-0.45553854	0.984540983
Tapbp	-0.698443354	-0.302464361	-0.324042215	1.40056602
H2-D1	-0.032926903	-0.262649115	-0.798000545	1.159238842
Gbp11	-0.80793338	-0.046461937	-0.420351994	1.286362795
Cfh	-1.191067322	0.265256324	-0.290231936	1.149728853
H2-K1	-0.306240348	-0.082280167	-0.781018273	1.190108829
Ccl12	-1.004903932	0.593960706	-0.389480243	0.651933293
Gbp3	-0.897573836	-0.214434958	-0.033629546	1.19924708
Ifi35	-0.42385418	0.065662429	-0.663202973	1.004979116
Ogfr	0.088939037	-0.219740817	-0.837073572	1.022810556
Gbp9	-0.926091793	0.000819809	-0.104334071	1.029401103
H2-T24	-0.9473801	-0.099098134	-0.114979913	1.186232681
Stat1	-1.107023769	0.188679835	-0.135316764	1.006490739
Samhd1	-1.041329439	-0.097062386	-0.100501722	1.263159143
Rtp4	-0.589390419	0.024474716	-0.392258377	0.951055401
Psmb10	-0.643543641	-0.18265173	-0.362240097	1.2340984
Ifit3	-0.537485241	0.074123434	-0.562458306	1.007289254
Herc6	-0.870001715	0.099843885	-0.226363464	0.971560322
Stat2	-0.823608594	0.032630688	-0.215853625	0.998673859
Gbp8	-1.25546677	0.005157417	0.226652366	1.022367632
Nod1	-0.989945904	-0.054903432	-0.142309285	1.200884479
Psme2	-0.641474752	-0.236138307	-0.279782581	1.216430218
H2-Q7	-0.559592029	-0.753687894	0.456017339	1.045684557

Ccl11	-0.829963781	-0.011522506	-0.252487156	1.096854069
Il18bp	-1.137472106	-0.339751393	0.574272463	0.987888885
Sod2	-0.843019945	-0.326736798	-0.051083869	1.302524811
Slamf7	-1.053341913	-0.180599787	0.280661628	0.998430019
Lgals3bp	-0.736759135	-0.430531701	0.052596575	1.222327187
Nlrc5	-0.919999271	-0.177793491	-0.048416324	1.190657458
Itgb2	-0.810288352	-0.528287513	0.76441746	0.706230282
Batf2	-0.977194773	-0.342485077	0.187607824	1.217693296
Gbp5	-1.100775107	-0.032995756	0.013625098	1.128394704
Trafd1	-0.680145373	-0.40812266	-0.067096329	1.257395027
Znfx1	-1.148558655	0.249802431	-0.167138931	1.003444548
Stat3	-1.017802309	0.431823266	-0.457834987	0.935858213
Ly6e	-1.179933537	0.14963086	0.06782009	0.925074872
Mt2	-0.82771492	0.648976951	-0.536745258	0.553238989
Ptgs2	-0.812524328	-0.160797334	0.864648784	0.148872211
Casp4	-0.544367518	-0.468247582	0.105273918	1.024403078
Gbp2	-1.042685679	-0.16252294	0.080070668	1.165768686
Fpr2	-0.531009757	-0.713208841	0.688276092	0.734244716
Mvp	-0.245397646	-0.122212959	-0.679296292	1.077460137
Ifih1	-0.887938313	0.065625833	-0.152881997	0.958788019
Il15ra	-0.817781737	-0.399450883	0.18982599	1.127269351
Wars	-0.55759426	-0.657983817	0.148776031	1.231298
Irf2	-1.063015862	0.288974934	-0.061422152	0.763219346
Cd274	-0.849170617	-0.46720303	0.336602249	1.096572156
Psmb8	0.045019486	-0.38346523	-0.500876285	0.935188336
Sp110	-1.168761809	0.20095753	-0.047340255	0.964905152
Ccl5	-0.95582644	-0.537507157	0.736113943	0.891596443
Gch1	-1.019353503	-0.18236899	0.281681113	0.965633628
Il4ra	-0.652316204	-0.676160993	0.57784911	0.919668335
H2-Q6	-0.554133319	-0.725416854	0.472033895	0.988870492
B2m	-1.1003868	0.382371841	-0.061227769	0.683649768
Cd38	-0.71885682	-0.539736599	0.767827758	0.625699811
Parp12	-0.582770449	-0.292120038	-0.29747235	1.245392846
Tnfaip3	-1.234336393	0.200278633	0.26258402	0.721404082
Ddx58	-1.045182834	0.240009777	0.002375255	0.742795358
Gbp4	-1.02615171	-0.101011517	0.211743972	0.940672133
Ccl7	-1.280786689	0.3994843	-0.020724617	0.802155931
Cflar	-1.104504259	0.308567474	0.11010531	0.608689607
Il15	-0.885133864	0.10261008	-0.268205946	1.025077211
Ube2l6	-1.00505253	0.210896321	-0.174042214	0.915474342
Trim21	-0.783785034	-0.028390242	-0.265696203	1.084969041
Casp12	-0.877666035	0.535691836	-0.405244591	0.613295831
Rnf31	-0.897979646	-0.326500126	0.282982387	1.023122416
Helz2	-0.510587799	-0.186172097	-0.320205027	1.063507946

Rnf213	-0.866596406	-0.0952769	-0.11360123	1.09929376
Vcam1	-1.059943002	0.504107883	-0.211666572	0.64147472
Fpr1	-0.506098992	-0.83422796	0.831607276	0.717276665
Mt1	-0.751117575	1.014246387	-0.821140181	0.304449772
Ifi27	-0.345001111	0.150762713	-0.726948059	0.883495779
Tnfsf10	-0.892841208	-0.548253951	0.911408639	0.666750008
Parp14	-0.909221225	-0.071850105	0.027399207	0.971634649
Epsti1	-0.829104417	-0.342766027	0.569959347	0.687602604
Fcgr1	-0.695981773	-0.637189516	0.899878555	0.592590113
Lysmd2	-1.06968529	-0.058264309	-0.051583547	1.194099223
H2-Q4	-0.110443212	-0.250999664	-0.614411156	1.038603948
Gbp7	-0.917554308	-0.007519144	0.033740461	0.893212776
Sppl2a	-1.067912498	0.59360038	-0.107070391	0.432982414
H2-Q10	-0.589064828	-0.331240613	0.126925124	0.87619047
Mean Score	-0.77956137	-0.088494967	-0.11762681	1.007806889

Table S4. List of enriched signature genes of the IFN-α response heat map.

gene_name	Control_Mean	Nintedanib_Mean	PD_L1_Mean	Combination_Mean
AC168977.1	-0.405240103	-0.01012377	-0.787854563	1.205749378
AC133103.1	-1.047997003	0.510245622	-0.571098436	0.98128841
H2-T22	-0.805996553	-0.065049204	-0.428396582	1.315704641
H2-T23	-0.635567103	-0.288505978	-0.375467674	1.37166725
Psmb9	-0.694521069	-0.062797968	-0.497838087	1.270856615
Tap1	-0.500941282	-0.393740957	-0.416591586	1.409709064
Bst2	-0.52444032	-0.138235268	-0.448201788	1.145436193
Mx2	-0.318862773	0.014705432	-0.667264422	0.967745405
Oas1b	-0.596328242	-0.090597251	-0.387803402	1.097378207
Ifi27l2a	-0.452940402	0.139426252	-0.723056307	1.001713895
Ifitm3	-0.689908139	0.044190217	-0.585159994	1.219830361
Dhx58	-0.576927393	0.03833996	-0.45553854	0.984540983
H2-D1	-0.032926903	-0.262649115	-0.798000545	1.159238842
Gbp11	-0.80793338	-0.046461937	-0.420351994	1.286362795
H2-K1	-0.306240348	-0.082280167	-0.781018273	1.190108829
Gbp3	-0.897573836	-0.214434958	-0.033629546	1.19924708
Ifi35	-0.42385418	0.065662429	-0.663202973	1.004979116
Ogfr	0.088939037	-0.219740817	-0.837073572	1.022810556
Gbp9	-0.926091793	0.000819809	-0.104334071	1.029401103
H2-T24	-0.9473801	-0.099098134	-0.114979913	1.186232681
Rtp4	-0.589390419	0.024474716	-0.392258377	0.951055401
Trim30d	-1.04588107	0.247105613	-0.239723888	0.976722941
Trim12a	-0.649825985	-0.100642789	-0.372757142	1.148386613
Ifit3	-0.537485241	0.074123434	-0.562458306	1.007289254
Herc6	-0.870001715	0.099843885	-0.226363464	0.971560322
Stat2	-0.823608594	0.032630688	-0.215853625	0.998673859
Gbp8	-1.25546677	0.005157417	0.226652366	1.022367632
Psme2	-0.641474752	-0.236138307	-0.279782581	1.216430218
H2-Q7	-0.559592029	-0.753687894	0.456017339	1.045684557
Lgals3bp	-0.736759135	-0.430531701	0.052596575	1.222327187
Uba7	-0.629220061	-0.383565663	-0.115785126	1.224462266
Batf2	-0.977194773	-0.342485077	0.187607824	1.217693296
Gbp5	-1.100775107	-0.032995756	0.013625098	1.128394704
Trafd1	-0.680145373	-0.40812266	-0.067096329	1.257395027
Csf1	-1.215287351	0.20269669	-0.025781295	0.987697784
Ly6e	-1.179933537	0.14963086	0.06782009	0.925074872
Sell	-0.912308784	-0.190795239	0.650615689	0.500187144
Gbp2	-1.042685679	-0.16252294	0.080070668	1.165768686
Ifih1	-0.887938313	0.065625833	-0.152881997	0.958788019
Wars	-0.55759426	-0.657983817	0.148776031	1.231298
Irf2	-1.063015862	0.288974934	-0.061422152	0.763219346

Psmb8	0.045019486	-0.38346523	-0.500876285	0.935188336
Sp110	-1.168761809	0.20095753	-0.047340255	0.964905152
Trim5	-0.944913823	0.077247175	-0.102313299	0.950668153
Il4ra	-0.652316204	-0.676160993	0.57784911	0.919668335
H2-Q6	-0.554133319	-0.725416854	0.472033895	0.988870492
Trim12c	-0.852267387	0.095568272	-0.190979417	0.923786463
B2m	-1.1003868	0.382371841	-0.061227769	0.683649768
Parp12	-0.582770449	-0.292120038	-0.29747235	1.245392846
Gbp4	-1.02615171	-0.101011517	0.211743972	0.940672133
Cflar	-1.104504259	0.308567474	0.11010531	0.608689607
Il15	-0.885133864	0.10261008	-0.268205946	1.025077211
Ube2l6	-1.00505253	0.210896321	-0.174042214	0.915474342
Trim21	-0.783785034	-0.028390242	-0.265696203	1.084969041
Rnf31	-0.897979646	-0.326500126	0.282982387	1.023122416
Trim30a	-1.010472585	0.119046087	0.020611346	0.84105363
Helz2	-0.510587799	-0.186172097	-0.320205027	1.063507946
Ifi27	-0.345001111	0.150762713	-0.726948059	0.883495779
Parp14	-0.909221225	-0.071850105	0.027399207	0.971634649
Epsti1	-0.829104417	-0.342766027	0.569959347	0.687602604
H2-Q4	-0.110443212	-0.250999664	-0.614411156	1.038603948
Gbp7	-0.917554308	-0.007519144	0.033740461	0.893212776
H2-Q10	-0.589064828	-0.331240613	0.126925124	0.87619047
Mean Score	-0.733220691	-0.091192361	-0.19145417	1.038665312
